# Therapeutic Potential of Hydrogen Sulfide in Reproductive System Disorders

**DOI:** 10.3390/biom14050540

**Published:** 2024-04-30

**Authors:** Xutao Sun, Caiyun Mao, Ying Xie, Qing Zhong, Rong Zhang, Deyou Jiang, Yunjia Song

**Affiliations:** 1 Department of Typhoid, School of Basic Medical Sciences, Heilongjiang University of Chinese Medicine, No. 24, Heping Street, Harbin 150040, China; sunxutao@hljucm.net; 2 Department of Pharmacology, School of Basic Medical Sciences, Heilongjiang University of Chinese Medicine, No. 24, Heping Street, Harbin 150040, China; 17737493595@163.com (C.M.); zqq3364@163.com (Q.Z.); rongzhang202110@163.com (R.Z.); 3 Department of Synopsis of the Golden Chamber, School of Basic Medical Sciences, Heilongjiang University of Chinese Medicine, No. 24, Heping Street, Harbin 150040, China; xieying@hljucm.edu.cn

**Keywords:** H_2_S, reproductive system, disorders, oxidative stress

## Abstract

Hydrogen sulfide (H_2_S), previously regarded as a toxic exhaust and atmospheric pollutant, has emerged as the third gaseous signaling molecule following nitric oxide (NO) and carbon monoxide (CO). Recent research has revealed significant biological effects of H_2_S in a variety of systems, such as the nervous, cardiovascular, and digestive systems. Additionally, H_2_S has been found to impact reproductive system function and may have therapeutic implications for reproductive disorders. This paper explores the relationship between H_2_S and male reproductive disorders, specifically erectile dysfunction, prostate cancer, male infertility, and testicular damage. Additionally, it examines the impact of H_2_S regulation on the pathophysiology of the female reproductive system, including improvements in preterm birth, endometriosis, pre-eclampsia, fetal growth restriction, unexplained recurrent spontaneous abortion, placental oxidative damage, embryo implantation, recovery of myometrium post-delivery, and ovulation. The study delves into the regulatory functions of H_2_S within the reproductive systems of both genders, including its impact on the NO/cGMP pathway, the activation of K^+^ channels, and the relaxation mechanism of the spongy smooth muscle through the ROCK pathway, aiming to broaden the scope of potential therapeutic strategies for treating reproductive system disorders in clinical settings.

## 1. Introduction

The reproductive system, as one of the most important components of the human body, is mainly divided into the male and female reproductive systems and is an important basis for human survival and reproduction. The organs of the reproductive system are categorized according to their location, including the penis, testicles, prostate gland, vas deferens, and epididymis in males [1], whereas women are mainly composed of the fallopian tubes, ovaries, uterus, and vagina [2]. The structurally intact and normal function of the reproductive system of both sexes is essential for the health and reproduction of both sexes. Environmental contaminants, microorganisms, and poor lifestyles can lead to structural damage or dysfunction of the reproductive system [3,4,5,6,7,8]. In addition, it is also associated with various reproductive disorders such as decreased fertility, erectile dysfunction (ED), varicocele, preterm labor, pre-eclampsia, and fetal growth restriction. Due to the complexity and diversity of the pathogenesis of diseases of the reproductive system, there are no clear and effective measures to stop the progression of the diseases. Therefore, there is an urgent need to address this issue and to identify possible diagnostic and therapeutic targets for related diseases.

Hydrogen sulfide (H_2_S) is the third endogenous gaseous signal molecule discovered after nitric oxide (NO) and carbon monoxide (CO). It is involved in the regulation of multiple organ systems, including the reproductive system [9,10], and biological processes such as angiogenesis, inflammation, oxidative stress, autophagy, and apoptosis [11,12,13]. H_2_S generation systems have been found in the mammal reproductive systems [14,15,16]. With regard to its role in the male reproductive system, H_2_S promotes the relaxation of human cavernous smooth muscle, mediates erectile function [17,18,19,20], enhances spermatogonial proliferation [21,22], and regulates the relaxation of vas deferens smooth muscle [23,24]. Among the effects of H_2_S on the female reproductive system, H_2_S suppresses the natural contraction of uterine bands taken from pregnant rats [25], notably relaxes vaginal smooth muscle in rabbits [16], modulates oviductal transport [26], and causes placental metamorphosis [27]. Altogether, H_2_S is significant in the reproductive systems of both men and women and could potentially be beneficial in treating reproductive system disorders. This review provides a comprehensive summary of the distribution, role, and mechanisms of H_2_S in the male and female reproductive systems, with the aim of providing a theoretical basis for the discovery of new therapeutic targets and the development of more effective therapeutic strategies for reproductive system diseases.

## 2. Physical and Chemical Properties of H_2_S

**Figure 1:**
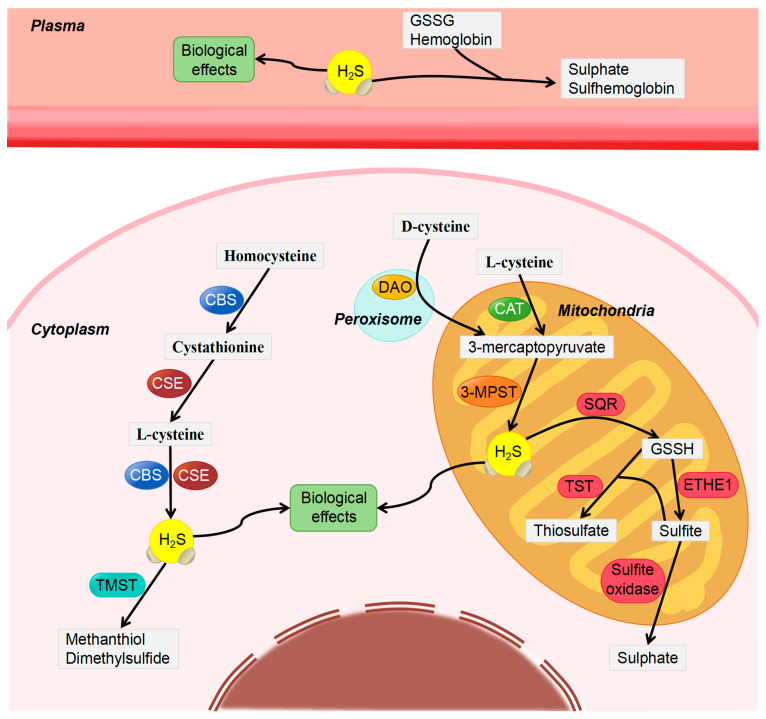
Generation and metabolism of endogenous H_2_S.

H_2_S is a small-molecule gasotransmitter that is highly lipophilic and can, therefore, penetrate cell membranes without the aid of specific transporter proteins or receptors. In the human body, H_2_S exists mainly as a hydrosulfide ion (HS-) and, to a lesser extent, as a free gas. Through a sequence of reactions, it is possible for it to undergo oxidation and produce sulfur dioxide, sulfates, and elemental sulfur, as well as various other substances [28,29]. A dynamic equilibrium is maintained between H_2_S and HS to ensure the stability of H_2_S in vivo and maintain the normal pH of the internal environment. In mammals, endogenous H_2_S is produced through desulfurization of cysteine, a process that involves three major tissue-specific enzymes, namely, cystathionine-β-synthase (CBS), cystathionine gamma-lyase (CSE), and 3-mercaptopyruvate sulfurtransferase (3-MPST) [30,31]. Both CBS and CSE are localized in the cytoplasm, whereas 3-MPST is mainly localized in mitochondria [32,33]. Endogenous H_2_S is generated from L-cysteine (L-Cys) and homocysteine by CBS and CSE [34,35]. In addition, it can be generated from 3-mercaptopyruvate by 3-MPST. 3-mercaptopyruvate is produced from either L-Cys (CAT/MPST pathway) by cysteine aminotransferase (CAT) or D-cysteine (DAO/MPST pathway) by amino acid oxidase (DAO) [36,37]. H_2_S is metabolized through the following three mechanisms in vivo: (1) Mitochondrial oxidation: H_2_S is first catalyzed by sulfide quinone oxidoreductase (SQR) at the inner mitochondrial membrane to form glutathione persulfide (GSSH), which is then oxidized to sulfite by persulfide dioxygenase (ETHE1) in the mitochondrial matrix. Sulfite can be further oxidized to sulfate by sulfite oxidase or reacted with GSSH to form thiosulfate under the transsulfuration of thiosulfate:cyanide sulfurtransferase (TST, rhodanese) [38,39]; (2) Cytoplasmic methylation: H_2_S is metabolized in the cytoplasm by thiol-S-methyltransferase (TMST) to methyl mercaptan, which is subsequently converted to dimethyl sulfide [40,41]; (3) Plasma molecule binding: Free H_2_S in plasma can bind to glutathione disulfide (GSSG) or other metal- or disulfide-containing molecules, such as hemoglobin, to form sulfate or H_2_S conjugates such as sulfhemoglobin (see Figure 1) [42,43]. The metabolite excretion occurs primarily via the kidney, spleen, and lungs. In the body, H_2_S levels remain controversial. The estimated physiological levels of circulating H_2_S range from 10 to 100 μM [44], whereas the concentration of peripheral H_2_S is estimated to be 30–100 μM [45], which may be lower in the reproductive system, with only nanomolar levels in the corpus cavernosum [46].

## 3. Distribution of H_2_S Synthases in the Reproductive System

### 3.1. Distribution of H_2_S Synthases in the Male Reproductive System

**Figure 2:**
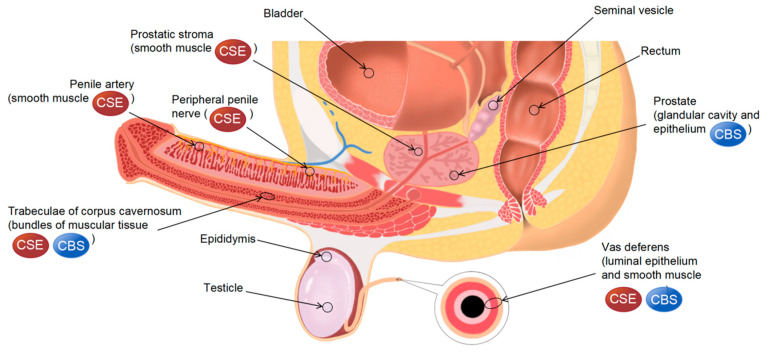
Distribution of H_2_S Synthases in the Male Reproductive System.

H_2_S generated within the male reproductive system was first identified in rabbit cavernous smooth muscle [47]. Shortly thereafter, d’Emmanuele et al. [17] demonstrated that CBS and CSE were found to produce endogenous H_2_S in the human corpus cavernosum (HCC) and identified the location of the two enzymes via immunohistochemical analysis of tissues. CSE was found in peripheral nerves and vascular smooth muscle cells (SMCs) of the penile and trabecular muscular tissue of HCC, while CBS was primarily found in trabecular muscular tissue. Both CBS and CSE were determined in the human prostate, with CBS substantially localized in the lumen and epithelial cells and CSE in the periacinar stroma [48,49]. The species and tissue distribution of H_2_S synthases expressed in other mammals, such as rats and mice, differ slightly from those in humans (Figure 2). In rat penile tissue, cavernous smooth muscle cells (CCSMCs) express CSE, CBS, CAT, DAO, and 3-MPST but lack the expression of CBS [50,51]. CBS, CSE, and 3-MPST are predominantly expressed in the mouse corpus cavernosum, especially in luminal endothelial cells and vascular endothelial cells. However, immunohistochemical findings have shown that the expression of CSE is higher than that of CBS and 3-MPST [52]. In addition, only CBS is expressed in the luminal subcutaneous smooth muscle, vascular smooth muscle, and peripheral nerves of the corpus cavernosum [53]. In a study, five enzymes that produce H_2_S were found in the prostates of rats. CBS, MPST, and CAT were mainly localized in glandular epithelial cells. In contrast to MPST expression, CBS expression was significantly higher in PR-V than in PR-D, whereas CAT expression was not significantly different between the two sites [36]. CSE is essential for H_2_S synthesis in the mouse prostate, with its expression decreasing with age [54].

To date, most studies investigating the distribution of H_2_S in the testis have used animal models. In rats, CBS is mainly discovered in the interstitial cells of seminiferous tubules. And it is moderately expressed in immature germ cells in the marginal region of seminiferous tubules but rarely expressed in mature germ cells. CSE is abundant in the vascular wall of the testicular interstitium, supporting cells, and immature germ cells [21]. In mice, three H_2_S-generating enzymes are expressed in testicular germ cells [55]. In humans, mice, and rats, CSE and CBS are mainly localized in the luminal epithelium and muscular tissue of the vas deferens [23]. In rats, CBS and CSE mRNA levels are higher in the somatic and caudal regions of the epididymis, with CBS identified in epithelial cells and CSE in SMCs below the epithelium [56].

### 3.2. Distribution of H_2_S Synthases in the Female Reproductive System

**Figure 3:**
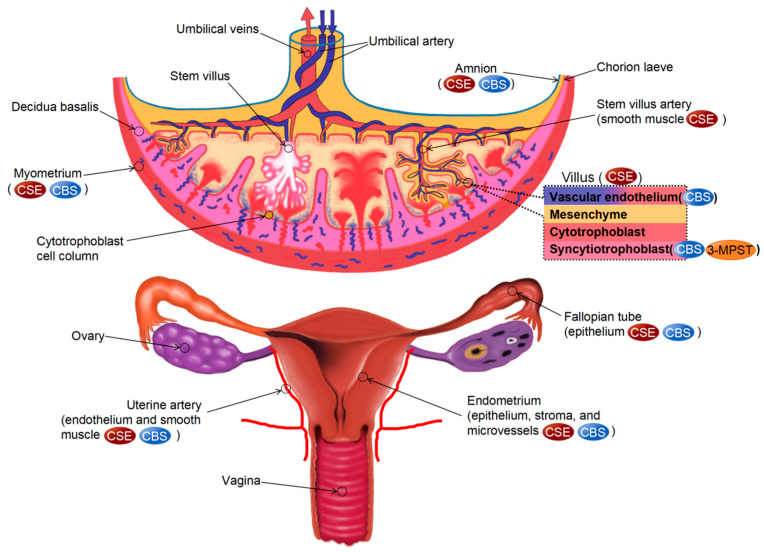
Distribution of H_2_S synthases in the female reproductive system.

Patel et al. [57] were the first to examine the production of endogenous H_2_S and the distribution of CBS and CSE in human and rat uterine tissues. They qualitatively detected the expression of CBS and CES in the non-pregnant uterus, pregnant uterus, placenta, and fetal membranes of rats and the chorionic villi, amnion, and myometrium of the human placenta using protein blotting. Subsequent studies have indicated that 3-MPST is present in the human placenta, with CBS and CSE predominantly found in syncytiotrophoblasts and vascular endothelial cells in the chorionic villi. Meanwhile, 3-MPST was observed in syncytiotrophoblasts [58,59]. In addition, CSE is expressed in trophoblasts and mesenchymal cells within the chorionic core of human placental villi and in the SMCs of chorionic trunk arteries [60,61]. Unlike the expression of CBS and CSE, the expression of 3-MPST in placental tissues does not show a significant difference between a healthy female and a female with pre-eclampsia [59]. A similar expression of CBS and CSE is observed in the endothelial cells and SMCs of human uterine arteries [62,63]. In addition, 3-MPST and CAT are expressed in human uterine arteries. The levels of CBS mRNA and protein are increased during gestation and the proliferating stage of the menstrual cycle; however, CSE, 3-MPST, and CAT protein expression do not undergo significant changes during these periods [62]. In human endometrium, the expression of CBS is upregulated during these two periods, whereas that of CSE is not significantly altered. CBS and CSE are primarily found in the epithelium, stroma, and microvessels within the human endometrium. However, CBS is specifically located in stromal cells and blood vessels during pregnancy and the proliferative stage of the menstrual cycle [64]. In mouse ovaries, CBS is expressed in follicular cells at all stages, with its expression being higher in primary and primordial follicles than in secondary and sinus follicles. After follicular development, granulosa cells are divided into cumulus cells and mural granulosa cells, and high levels of CBS are observed in both subpopulations. However, CBS is not found in oocytes [15]. In human fallopian tubes, CBS and CSE are determined in the tubal epithelium [26]. Similarly, both enzymes are expressed in human vaginal tissues. Sun et al. [65] found that CBS and CSE are predominantly discovered in rat vaginal epithelial cells (VK2/E6E7), and CSE was expressed in rat vaginal vessels by immunohistochemical analysis (Figure 3).

## 4. Effects of H_2_S on Male Reproductive System Disorders

### 4.1. H_2_S Promotes Penile Erection

#### 4.1.1. H_2_S Relieves Erectile Dysfunction

Penile erection relies mainly on smooth muscle relaxation in the corpus cavernosum and penile arteries for its morphology and function. Upon stimulation, parasympathetic nerves release neurotransmitters that relax the smooth muscles in the corpus cavernosum and penile arteries. In response to this relaxation, blood flows into the cavernous sinus, causing it to swell and compress the veins, thereby reducing blood outflow and raising intracavernous pressure (ICP), which ultimately results in an erection [66]. Several studies have demonstrated that H_2_S promotes penile erection. Jupiter et al. [67] reported that the H_2_S donors Na_2_S and NaHS promoted penile erection and prolonged the duration of erection in anesthetized rats in a concentration-dependent manner. According to Srilatha et al. [18], injecting NaHS into non-human primates’ cavernous bodies notably elevated penile length and ICP. These changes were consistent with those observed before penile erection was induced by the injection of prostaglandin E1. Another study showed that injection of the CSE inhibitor DL-propargylglycine (PAG) into the external jugular vein of rats inhibited H_2_S production, consequently decreasing ICP induced by the electrical stimulation of cavernous nerves. It was also shown that injecting rats with PAG, an inhibitor of CSE, to suppress H_2_S generation reduced electrical stimulation and cavernous nerve-induced ICP. This finding suggests that cavernous nerve excitation mediates penile erection by facilitating the release of H_2_S. Consistently, d’Emmanuele et al. [17] demonstrated the presence of a CSE/H_2_S-generating system in the peripheral nerves of the human penis.

The erection of the penis is regulated by nerves, blood vessels, and smooth muscles. An injury to one or more parts of the penis can result in erectile dysfunction (ED). It has been determined that rats with ED due to radical prostatectomy, hyperlipidemia, diabetes, or hypertension have reduced levels of H_2_S-generating enzyme in their penile tissues [19,51,68,69]. Furthermore, La Fuente et al. reported that ED is associated with defects in the L-cysteine/H_2_S pathway [70]. However, supplementation with exogenous H_2_S donors can alleviate ED. ACS6 was the first drug, developed from Sildenafil and ADT-OH, able to supplement H_2_S [20]. While ACS6 is similar to sildenafil in terms of relaxing the corpus cavernosum at the same dose, its potency in suppressing superoxide formation and PDE5 expression is higher than that of sildenafil citrate or NaHS. Notably, treatment with ACS6 for a period could ameliorate ED through suppressing oxidative stress and decreasing PDE5 levels. There is evidence that certain extractions from plants stimulate H_2_S generation in vivo. For instance, resveratrol (RVT) [71] induces the relaxation of corpus cavernosum (CC) in mice in a concentration-dependent manner. Inhibitors of CBS counteract RVT’s effects, but not those of eNOS. In response to RVT, the production of H_2_S is increased at both basal and L-cysteine-induced levels. It appears that RVT-stimulated CC relaxation is partly dependent on H_2_S production and is not dependent on NO production. Furthermore, it was discovered that sodium tanshinone IIA sulfonate (STS) [69] reversed the downregulation of CBS and CSE and inhibited H_2_S production in rats fed a high-fat diet (HFD). Additionally, STS guarded against ED through the activation of the Nrf2/HO-1 pathway in response to HFD-induced oxidative stress.

In regards to treating ED, preferred medications such as sildenafil and tadalafil (also known as PDE-5is) are available [72]; however, their efficacy is poor in some cases. β3 adrenergic receptors are found in the SMCs of HCC. Upon activation, these receptors result in smooth muscle relaxation in HCC under the influence of cGMP [73]. An activator of the β3 adrenergic receptor, BRL37344, was discovered to relax HCC and the penile artery, whereas PAG reversed these relaxation effects [74]. Treatment of both HCC bands and tissue homogenates from penile arterial rings with BRL37344 significantly increased H_2_S production; however, inhibition of H_2_S generation prevented the BRL37344-mediated increment of cGMP level in HCC and penile arterial tissues. These results suggest that activation of β3 adrenoceptors leads to relaxation of HCC and penile arteries in an H_2_S/cGMP-dependent manner. Given that selective agonists of β3 adrenoceptors, such as mirabegron [75], function independently of the endothelium, they can be used as replacement medications for these individuals who have no effect on PDE-5 therapy.

Because H_2_S is mainly released by SMCs and is independent of endothelial function, it represents a potential treatment option for ED related to endothelial malfunction, such as ED related to diabetes and metabolic syndrome (metS). High-glucose diet-induced metS has been shown to reduce H_2_S production in rat penile tissues [76]. However, exogenous administration of H_2_S donors can improve erectile function. A study showed that GYY4137, a slow-releasing H_2_S donor, significantly improved the CC vascular responsiveness by restraining the TGF-β1/Smad/CTGF signal pathway in STZ-mediated diabetic rats [77]. Furthermore, H_2_S donors combined with PDE-5is hold potential applications for treating ED. Combining NaHS and tadalafil therapy was more effective than monotherapy against ED in some rat models of bladder outlet obstruction [78]. The reduced erectile response and decreased H_2_S levels were only partially restored after monotherapy but were completely restored after combination therapy. In addition, combination therapy counteracted changes in the morphology and function of the penis triggered by bladder outlet blockage-induced ischemia and improved the erectile response. These results suggest that H_2_S donors can alleviate ED and restore the spontaneous erectile response with long-term use.

#### 4.1.2. Mechanisms of H_2_S in Regulating Penile Erection

**Figure 4:**
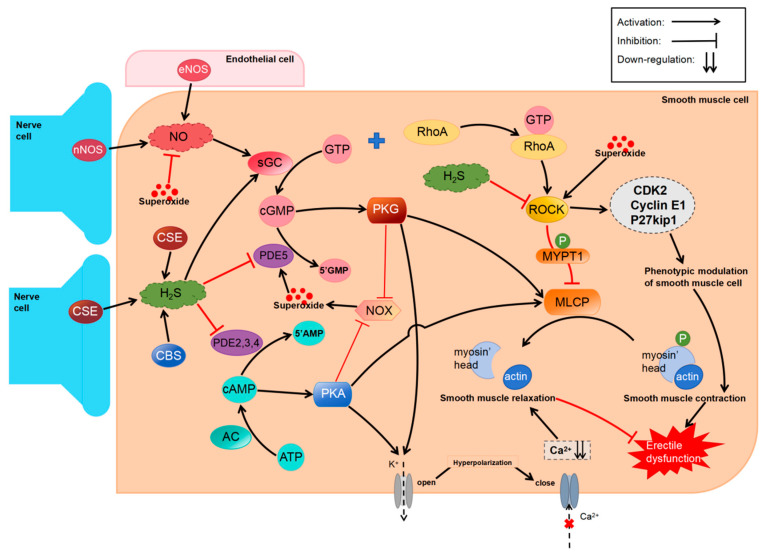
Mechanisms of H_2_S relieve erectile dysfunction.

Priapism is caused and maintained by more arterial blood inflow than venous return. Vascular smooth muscle relaxation plays a key role in priapism. Although the exact mechanism through which H_2_S relaxes CC smooth muscle remains unclear, it has been reported to involve the synergetic action of NO, stimulation of K^+^ channels, as well as the modulation of RhoA/ROCK (Table 1), with NO playing the most controvertible role. Penile erection is thought to be mediated primarily by NO [79]. It promotes the production of cGMP, an intracellular second messenger, by activating soluble guanylate cyclase (sGC), which in turn relaxes corpus cavernosum smooth muscle by regulating calcium channels and intracellular contractile proteins [80]. Several studies have shown that H_2_S can increase the expression of NOS, the key enzyme for NO production. Meng et al. [81] showed that eNOS activity, protein and mRNA expression, and NO levels were higher in rat cavernosum tissues treated with NaHS (1 mM) than in control tissues. Yilmaz et al. [68] found that administration of NaHS (0.037mg/kg) counteracted the decrease in eNOS and nNOS protein levels in penile tissues. Mostafa et al. [82] found that NaHS (30 mg/kg) increased the levels of NO in the cavernous tissues of rats, which is consistent with the aforementioned findings. However, knockout of CSE significantly reduced the levels of p-eNOS and NO in rat corpus cavernosum tissues [83] (Figure 4). These findings suggest that H_2_S regulates penile erectile function by promoting the production of NO.

Some in vivo studies have shown that NO, stimulation of K^+^ channels, and modulation of the RhoA/ROCK pathway do not have synergistic effects. Srilatha et al. [47] pre-contracted isolated rabbit CC bands with norepinephrine and L-NAME, an inhibitor of the NO-generating enzymes, followed by NaHS treatment. The relaxation caused by NaHS was not affected by L-NAME. In another study, cavernous smooth muscle was precontracted in the presence of atropine and then exposed to the CBS inhibitor aminooxyacetic acid (AOAA) or the CSE inhibitor β-cyanoalanine (BCA) or PAG, followed by electrical stimulation. The results showed that inhibition of H_2_S synthesis did not affect the NO-mediated relaxation of cavernous smooth muscle. Similarly, treatment with L-NAME did not cause a notable reduction in the relaxation of human corpus cavernosum smooth muscle induced by NaHS (1 μM–10 mM) [17]. The intracavernosal administration of Na_2_S (0.03–1 mg/kg) significantly increased ICP, whereas the intracavernosal administration of L-NAME did not significantly affect it [67]. Furthermore, intracavernosal administration of Na_2_S did not affect the erectile response caused by SNP. Mice lacking NO exhibit a higher level of CSE and 3-MPST, thereby increasing the production of H_2_S and its relaxation effects on the CC [52]. These changes may be compensatory but suggest that the relaxation effects of H_2_S are independent of NO. Therefore, H_2_S may not synergize with NO to promote the erectile response.

These conflicting results indicate that the mechanism by which the relaxation effects of H_2_S on CC smooth muscles in vivo may not be dependent on the NO-cGMP pathway. However, endogenous H_2_S inhibits phosphodiesterase (PDE) to suppress cGMP degradation [113]. Several studies have shown that H_2_S in the penis regulates cGMP levels by acting on sGC. For example, treatment with L-Cys (1 μM–1 mM) and NaHS (1 μM–1 mM) increases cGMP levels in HCC, whereas administration with ODQ, an inhibitor of sGC, reverses this effect [114]. The decrease in H_2_S content in CSE-KO mice weakens the redox state of sGC, thereby downregulating cGMP in the penis [83], suggesting that H_2_S promotes penile erection by upregulating cGMP through other pathways.

Potassium channels have been reported to participate in the H_2_S-mediated relaxation of corpus cavernosum smooth muscle. There are four basic types of K^+^ channels exhibited in arterial smooth muscle: KCa, K_ATP_, Kir, and Kv channels [115]. These four channels are also found in human corpus cavernosum tissues [116]. To examine the role of different K^+^ channels in the H_2_S-mediated relaxation of corpus cavernosum smooth muscle, Jupiter et al. [67] injected tetraethylammonium chloride (TEA, a non-selective inhibitor of K^+^ channels), iberiotoxin (an inhibitor of BKCa channels), and glyburide (GLB, an inhibitor of K_ATP_ channels) into the cavernous bodies of anesthetized rats. These three compounds affected the changes in ICP induced by Na_2_S (0.03–1 mg/kg). Intracavernous administration of Na_2_S increased ICP, which was counteracted by TEA and iberiotoxin administration; however, the effects of GLB on ICP were not evident. In another study, rat CC bands were treated with TEA, GLB, 4-AP, a Kv channel inhibitor, or BaCl2, a Kir channel inhibitor, in an organ bath, and the effects of these compounds on the CC relaxation caused by L-cysteine were also examined. According to the results, TEA and 4-AP notably weakened the L-cysteine-stimulated CC relaxation effect, with TEA being more effective than 4-AP. However, GLB and BaCl2 did not have significant inhibitory effects on L-cysteine-induced relaxation [84]. The above results indicated that BKCa and Kv channels are associated with H_2_S-induced CC relaxation in the rat. GYY4137 relaxes rat CC partially via KATP channels [85]. Another study showed that GLB remarkably attenuated the NaHS-induced relaxation of HCC bands [17], while, as for the concentration of GLB used in the experiment, it is more than 10 times and may have interfered with the results since GLB at more than 10 times suppresses Na^+^-K^+^ pumps and L-type Ca^2+^ channels [117]. Correspondingly, the researchers found that administration of GLB at 10 μM did not inhibit NaHS-mediated relaxation of CC strips in rats [50,84] or humans [114].

ROCK, a serine/threonine kinase that promotes smooth muscle contraction, is involved in the H_2_S-mediated relaxation of cavernous smooth muscle. Chitaley et al. [118] found that treatment with Y-27632, an inhibitor of ROCK, increased ICP and stimulated penile erection in rats. These effects were found to be independent of the NO pathway. It was found that NaHS had significantly greater relaxation effects on HCC strips precontracted with the ROCK modulator U46619 or endothelin-1 than phenylephrine on HCC strips [17], implying that H_2_S may regulate the ROCK pathway. A ROCK inhibitor, Fasudil, can also inhibit the relaxation effects of H_2_S donors on mouse CC, indicating that H_2_S may interact with ROCK [119]. Aydinoglu et al. [86] reported for the first time that ROCK was involved in the H_2_S-induced relaxation of the corpus cavernosum of mice contracted with phenylephrine. Pretreatment with Y-27632 significantly attenuated the contractile effects induced by phenylephrine-phosphorylating MYPT1 at Thr696 in isolated mouse CC strips. In addition, treatment with L-cysteine or NaHS substantially eliminated the contractile response of phenylephrine. Combining Y-27632 with L-cysteine or NaHS decreased MYPT1 phosphorylation, while PAG or AOAA reversed the inhibition. Additionally, Y-27632 notably elevated H_2_S generation at basic levels and in response to L-cysteine, whereas PAG and AOAA inhibited H_2_S synthesis, indicating that ROCK may at least partially inhibit CSE and CBS levels in CC tissues [86]. The RhoA/ROCK pathway can promote CC smooth muscle contraction by modulating the CCSMCs phenotype [19]. Researchers have concluded that in rats with bilateral cavernous nerve damage, treatment with NaHS suppressed the phenotypic conversion of CCSMCs caused by the enhancement of the RhoA/ROCK pathway, contributing to the improvement of erectile dysfunction [19].

### 4.2. Regulatory Role of H_2_S in Prostate Cancer

The effects of H_2_S on the prostate are mainly examined in the context of prostate cancer (PCa), especially castration-resistant prostate cancer (CRPC). The growth and development of the prostate rely on androgens. At present, one of the most widely used hormone therapies involves the direct blockade of the androgen receptor (AR). H_2_S was found to inhibit the trans-activation of the second zinc finger of AR through S-sulfhydration of cysteine at residues 611 and 614 and effectively suppress the growth of PCa cells resistant to anti-androgen therapy [54]. Bicalutamide is an anticancer agent that competitively binds to AR. It was found that CSE expression was substantially lower in bicalutamide-resistant PCa cells (LNCaP-B) than in androgen-dependent PCa cells (LNCaP). In addition, overexpression of CSE or exogenous administration of H_2_S (30 μM NaHS) restored the sensitivity of LNcap-B cells to bicalutamide, whereas LNCaP cells with knockdown of CSE continued to grow even in the presence of bicalutamide [54]. These results suggest that the CSE/H_2_S system can be used as a valuable prognostic indicator and an effective therapeutic target for early-stage PCa and CRPC. However, some studies have reported that H_2_S plays a negative regulatory role in PCa. It was shown that upregulation of cytoplasmic cAMP caused by androgen deprivation increased the generation of the CSE/H_2_S system [87]. Subsequently, H_2_S promoted androgen-independent proliferation by enhancing the activity of Cav3.2. Both endogenous and exogenous H_2_S can promote the function of T-type Ca^2+^ channels in LNCaP cells with neuroendocrine differentiation potential, consequently promoting the progression to neuroendocrine CRPC.

The above results suggest that the CSE/H_2_S system may be involved in the regulation of PCa progression. However, the opposite regulatory results may be because the response of cancer cells to H_2_S varies with the type of H_2_S donor, concentration of the donor, and type of cancer [120]. H_2_S-related drugs have been reported to exert good therapeutic effects against PCa. For example, H_2_S-releasing non-steroidal anti-inflammatory drugs (HS-NSAIDs), in which traditional NSAIDs are covalently attached to an H_2_S-releasing moiety, are 28–3000 times more effective than traditional NSAIDs in inhibiting tumor cell growth in multiple cancers, including PCa [121]. In the castration- and Adriamycin-resistant PCa cell line DU-145, the accumulation of H_2_S-releasing doxorubicin (H_2_SDox) is significantly higher than that of traditional doxorubicin. Mechanistically, the release of the SH2 group from H_2_SDox damages the activity of PCa cells by forming disulfide bonds on P-gp, consequently overcoming Adriamycin resistance [122]. Recently, a tumor microenvironment (TME)-responsive Zn^2+^-interference and H_2_S-mediated gas therapy based on tannic acid-modified zinc sulfide nanoparticles (ZnS@TA) was discovered. In a neutral environment (pH = 7.4), the same concentration of ZnS@TA had no significant effect on the viability of DU-145 cells but significantly inhibited their migratory and invasive abilities and enhanced their apoptosis in TME [123].

### 4.3. H_2_S Promotes Male Fertility

**Figure 5:**
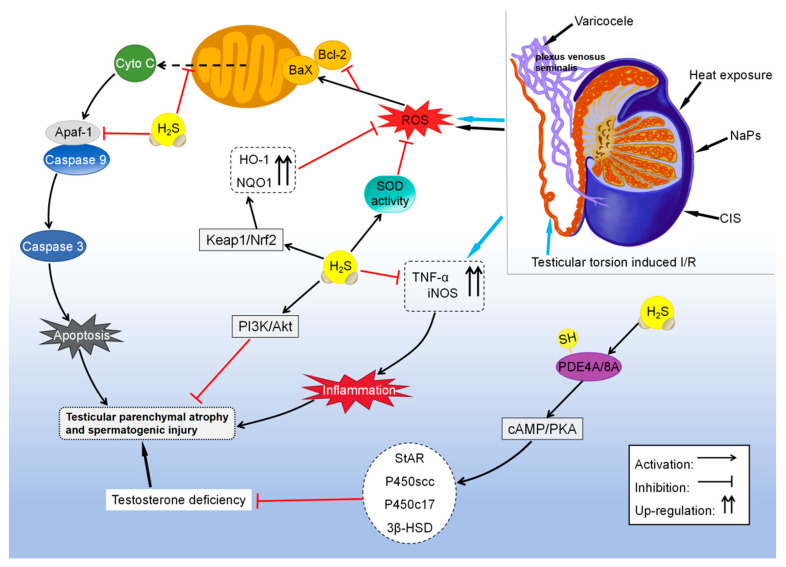
Mechanism of H_2_S protection against the testis and germ cells.

When combined with NH3, H_2_S in exhaust emissions can disrupt spermatogenesis [124] and reduce sperm motility through AMPK/AKT-related pathways [88], eventually impairing male fertility. However, many studies have shown that H_2_S protects the testis and sperm owing to its anti-inflammatory, antioxidative, and anti-apoptotic properties (Figure 5). Heat stress significantly increases the production of ROS and inhibits the activity of superoxide dismutase (SOD) in mouse germ cells. However, treatment with NaHS promotes SOD activity, reduces ROS production, inhibits the release of cytochrome C, and reduces the Bax-to-Bcl-2 ratio, thereby suppressing the apoptosis of testicular germ cells induced by heat exposure [55]. In addition, restraint stress can increase the levels of ROS and malondialdehyde (MDA) and decrease the levels of CBS, CSE, and 3-MPST in rat serum, suggesting that downregulated H_2_S plays a key role in male infertility [125].

H_2_S has been shown to alleviate testicular dysfunction and germ cell apoptosis caused by toxic substances. A study found that treatment with the antineoplastic drug cisplatin significantly increased MDA levels and decreased SOD activity in rat testicular tissues. However, administration of NaHS counteracted all biochemical, histological, and morphological changes induced by cisplatin [126]. The researchers found that nanoplastics induced ROS-dependent mitochondrial apoptosis and autophagy in GC-2spd(ts) cells derived from mouse spermatocytes, resulting in reproductive toxicity. H_2_S alleviated nanoplastic-induced reproductive toxicity by increasing the expression of antioxidant enzymes such as HO-1 and NQO1 through Keap1/Nrf2 signaling [89]. Testosterone deficiency can affect the normal morphological features and motility of sperm and impair male fertility [127]. It was shown that overexpression of CBS restored testosterone levels by promoting the S-sulfhydration of PDE4A and PDE8A and activating the cAMP/PKA pathway in mouse stromal tumor cell (MLTC-1) models of lipopolysaccharide (LPS)- and hydrogen peroxide (H_2_O_2_)-induced testosterone synthesis impairment [91].

Some studies have shown that sperm motility is decreased in animal models of defective H_2_S synthesis; however, exogenous H_2_S administration or CBS overexpression increases sperm motility in these models. Impaired spermatogenesis caused by stress is associated with decreased production of endogenous H_2_S [92]. During artificial insemination, freezing thawing may cause oxidative stress damage to sperm [128]. Studies have validated that H_2_S donors can protect sperm from oxidative stress damage in vitro, maintain sperm motility, and reduce acrosome loss [93]. Notably, high levels of H_2_S can reduce sperm migration [129]. On the contrary, the co-administration of low-concentration H_2_S donors and NO donors can enhance the forward motility of boar sperm and protect the integrity of the plasma membrane under oxidative stress [130]. It was found that H_2_S-releasing agents based on amino acids simulated the release of physiological levels of H_2_S in the presence of carbonic anhydrase without exerting harmful effects on cell function. In addition, the H_2_S-releasing agents enhanced the forward motility of sperm during short-term treatment, which may prolong the survival of sperm [131]. These results suggest that the regulatory effects of H_2_S on sperm motility in vitro may contribute to assisted reproduction.

In addition, the contraction of the epididymis and vas deferens promotes the expulsion of sperm. H_2_S has been shown to regulate the function of the vas deferens and epididymis. It relaxes the smooth muscle of the vas deferens in a concentration-dependent manner and participates in the regulation of tonic contraction of the vas deferens [23,24]. Li et al. [24] used NaHS to treat vas deferens strips pretreated with L-NAME, TEA, iberiotoxin, GLB, 2-aminoethoxydiphenylborate (2-APB, a transient receptor potential channel inhibitor), and apamin (a small conductance calcium-activated potassium channel blocker). The results showed that L-NAME, GLB, 2-APB, and apamin did not affect the response of the vas deferens to NaHS, whereas TEA and iberiotoxin counteracted the relaxation effects of NaHS, suggesting that H_2_S targets BKCa channels to regulate vas deferens relaxation. Furthermore, N-ethylmaleimide, a sulfhydryl alkylation compound that protects thiols from oxidation, prevented the NaHS-induced relaxation of the vas deferens smooth muscle. These findings suggest that H_2_S directly or indirectly regulates the activity of BKCa channels through S-sulfhydration, thereby relaxing vas deferens smooth muscle. The microenvironment of the epididymal lumen is conducive to maintaining sperm motility, and functionally mature sperm are stored in the tail of the epididymis before ejaculation to maintain their immobility [132]. H_2_S promotes K^+^ secretion in the rat epididymal epithelium by activating K_ATP_ and BKCa channels. High K^+^ concentrations in the luminal fluid of the cauda epididymis can inhibit sperm motility in a pH-independent manner. H_2_S promotes the formation of an epididymal luminal microenvironment with high K^+^ concentrations, thus maintaining the cauda epididymal sperm in a quiescent state before ejaculation [56]. These results suggest the involvement of H_2_S in ejaculation. Additional research could help in creating new treatment approaches for asthenospermia, spermatorrhea, and premature ejaculation.

### 4.4. H_2_S Relieves Testicular Damage

H_2_S has been discovered to suppress the expression of iNOS and TNF-α, an inflammatory cytokine, to reduce tissue damage caused by inflammation, consequently alleviating I/R injury in testicular torsion. In addition, H_2_S inhibited apoptosis by suppressing the expression of apoptotic protease activator factor-1 (Apaf-1) [90]. These findings suggest that H_2_S plays a protective role in testicular injury. Furthermore, H_2_S has been shown to alleviate varicocele-induced testicular and epididymal damage. It was found that the weight of the left testis and epididymis and the diameter and epithelial thickness of seminiferous tubules were significantly reduced in rat models of varicocele. Long-term administration of NaHS restored these parameters by reducing oxidative stress and apoptosis in the testis [133]. Similarly, GYY4137 has been shown to alleviate varicocele-induced ipsilateral epididymal injury by activating the PI3K/Akt pathway in rat models (Figure 5) [94]. In addition, the researchers found that co-treatment with testosterone and NaHS reduced the damage caused by varicocele in rats. Compared with long-term administration of NaHS, combination therapy reduced the duration of treatment and the administered dose [134].

## 5. Effects of H_2_S on the Female Reproductive System Diseases

### 5.1. H_2_S Relieves the Preterm Birth

#### 5.1.1. H_2_S Relieves Abnormal Uterine Contractions-Caused Preterm Birth

H_2_S participates in the regulation of uterine contractility by relaxing the myometrium. Sidhu et al. [25] found that both endogenous H_2_S-generated substrates L-Cys and exogenous H_2_S donors NaHS effectively reduced the spontaneous contractions of pregnant rat uterine muscles in a concentration-dependent manner in vitro. It has consistently been shown that H_2_S exposure during gestation from day 6 prolongs labor in Sprague-Dawley rats [135]. In pregnant humans and rats, it was demonstrated that administration of GYY4137 or NaHS prevented preterm birth by relieving spontaneous contractions in the myometrium as well as oxytocin stimulation [136].

Sildenafil, a selective inhibitor of phosphodiesterase type 5 (PDE5), relaxes the smooth muscles of the pregnant uterus; however, its exact mechanism of action remains unclear. Mitidieri et al. [137] showed that sildenafil significantly increased the production of H_2_S in the mouse uterus, whereas CSE inhibitors attenuated the effects of sildenafil. However, sildenafil did not increase H_2_S production in CSE^-/-^ mice. Likewise, CSE^-/-^ mice showed less inhibition of spontaneous uterine contractility by sildenafil. These results suggest that the CSE/H_2_S system regulates the effects of sildenafil and the contractility of the uterus in mice. Given the therapeutic effects of H_2_S against uterine contractility disorders, H_2_S can be used as a novel inhibitor of premature uterine contractions to prevent preterm birth [138].

#### 5.1.2. H_2_S Relieves Inflammation-Caused Preterm Birth

**Figure 6:**
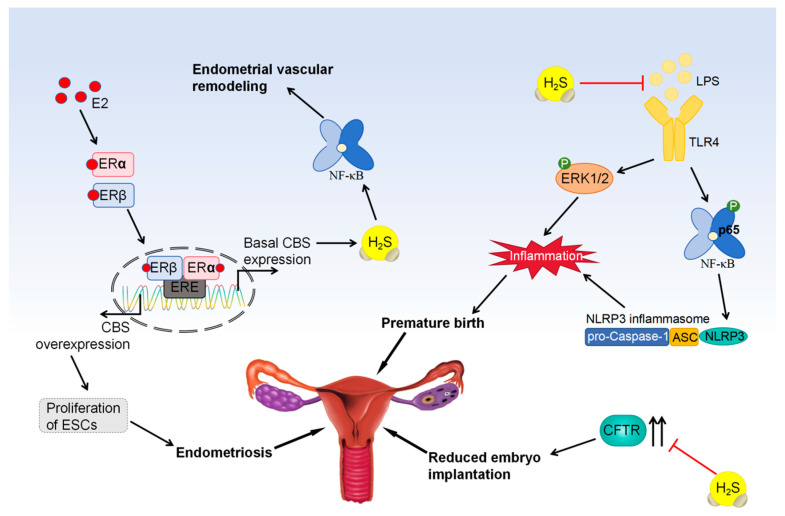
Regulatory mechanism of H_2_S in preterm birth and endometriosis.

Research conducted in the past has indicated that infection or inflammation is linked to 40% of premature births [139]. Activation of the maternal immune system results in elevated levels of white blood cells and inflammatory substances in the uterine muscle, which stimulate uterine contractions, cervical ripening, and rupture of the fetal membranes, ultimately causing premature birth [140]. Chen et al. [96] found that the expression of NLRP3, TLR4, and activated NF-κB was upregulated in mouse models of LPS-induced preterm uterine contractions. Treatment with NaHS decreased the expression of these three inflammatory factors and uterine constriction-associated protein (CAP) in a dose-dependent manner, consequently delaying LPS-induced preterm delivery in mice. However, treatment with TAK-242 (a TLR4 inhibitor) and BAY11-7082 (an NF-κB inhibitor) counteracted the increase in NLRP3 expression in human uterine SMCs treated with IL-1β. It is suggested that H_2_S suppresses the activation of NLRP3 inflammasomes through restraining the TLR4/NF-κB pathway, thereby maintaining the quiescent state of the uterus during infection or inflammation [97]. Upon binding to TLR4, LPS activates various signaling pathways, such as extracellular signal-activated kinase 1/2 (ERK1/2) and NF-κB [141]. Liu et al. [97] demonstrated that exposure to LPS led to elevated phosphorylated ERK1/2 and p65 levels in the myometrium, along with increased leukocyte infiltration into intrauterine tissue and upregulation of pro-inflammatory cytokines like IL-1β, IL-6, TNF-α, CCL2, and CXCL15. However, administration of NaHS counteracted the effects of LPS, indicating that H_2_S could be a potential focus for addressing infection-related preterm labor (Figure 6).

### 5.2. H_2_S Promotes the Endometrial Angiogenesis

During the proliferative phase of the menstrual cycle and during pregnancy, angiogenesis is responsible for the regeneration of the endometrium and the expansion of large blood vessels at the maternal-fetal interface [142,143]. Studies have shown that CBS expression and H_2_S production increase in the endometrium during pregnancy and the proliferative phase of the menstrual cycle. This increase is positively correlated with an increase in endogenous estrogen levels and endometrial angiogenesis during the two periods [64]. When the menstrual cycle is in the proliferative phase and pregnancy is in full swing, estrogen levels are remarkably high [144]. In ovariectomized nonpregnant ewes, estradiol (estradiol-17β, E2) replacement therapy enhances the CBS levels in the uterine artery endothelium and smooth muscle while having no effect on CSE expression [98]. In addition, E2 promotes CBS and CSE expression in ewe uterine artery SMCs (UASMCs) in a manner that depends on time and concentration. Notably, agonists that target specific estrogen receptors, namely, ERα or ERβ, can promote the expression of CBS and CSE in UASMCs [99]. Qi et al. [64] found that E2 increased the production of H_2_S by stimulating the estrogen receptor-dependent selective upregulation of CBS in endometrial stromal cells (ESCs). Nevertheless, the precise function of estrogen receptors in controlling the transcription of CBS is still not fully understood. Bai et al. [145] showed that E2 promoted CBS expression and H_2_S production in non-pregnant and pregnant uterine artery endothelial cells by activating the CBS initiation program. The binding of ERα and ERβ to estrogen response elements in the CBS promoter region plays an important role in this process. Therefore, ERα or ERβ activators can stimulate CBS/H_2_S production to levels comparable to E2-stimulated levels, whereas monotherapy with ERα or ERβ antagonists may block the E2-stimulated response. E2 has the ability to promote angiogenesis in endometrial microvascular endothelial cells (EMECs) when co-cultured with ESCs, leading to proliferation, migration, and tube formation. This phenomenon is validated by the higher potency of proliferating endometrium-derived ESCs than secretory endometrium-derived ESCs in stimulating the migration of EMECs. H_2_S donors can significantly stimulate angiogenesis in EMECs, whereas downregulation of CBS but not CSE inhibits the migration of EMECs [64]. Altogether, as the human endometrium proliferates and as pregnancy progresses, the CBS/H_2_S system is upregulated and participates in the regulation of estrogen during endometrial vascular remodeling. Various reproductive diseases, like endometriosis, are linked to the abnormal growth of blood vessels in the endometrium [146]. Research has revealed elevated levels of both CBS and CSE in the abnormal endometrial tissues of individuals and mice suffering from endometriosis [100]. However, inhibition of CBS and CSE notably reduced the number and weight of endometrial lesions in an allograft mouse model of peritoneal endometriosis. In vitro experiments showed that both exogenous and endogenous H_2_S promoted the proliferation of human ESCs, which was attenuated by inhibitors of CBS, CSE, or NF-κB (Figure 6). The findings indicate that H_2_S promotes the proliferation of ESCs via the triggering of the NF-κB pathway, offering valuable insights for the potential application of H_2_S inhibition in treating endometriosis.

### 5.3. H_2_S Promotes Embryo Implantation

The effects of H_2_S on uterine function are negligible. Dorman et al. [43] showed that continuous exposure to H_2_S (10–80 PPM, 6 h/d) before and after reproduction in female Sprague-Dawley rats (F0 generation) had no significant effect on pregnancy success rates, pregnancy process, or litter size. Guzman et al. [101] showed that the uterus of CBS-knockout pregnant mice underwent significant morphological changes and had decreased quality. Although the number of fertilized eggs implanted in the uterus did not decrease, the embryo survival rate decreased significantly. Notably, neither the ovaries nor the ovulatory oocytes of CBS-knockout mice showed significant morphological changes, and transplantation of CBS-null ovaries into mice with ovariectomizing mice fully restored fertility. These findings suggest that uterine dysfunction instead of ovarian dysfunction results in sterility in CBS-deficient female mice. The dynamic regulation of the uterine fluid environment required for embryo implantation is inseparable from the ion transport of the endometrial epithelium [147]. Cystic fibrosis transmembrane conductance regulator (CFTR) facilitates the release of Cl^-^ to promote the secretion of uterine fluid; however, its overexpression can lead to the formation of hydrops and hence prevent embryo implantation [148]. Xu et al. [107] showed that exogenous administration of H_2_S increased the concentration of Cl^-^ in mouse endometrial epithelial cells and the I^-^-dependent short-circuit current (ISC). A specific CFTR inhibitor, CFTRinh-172, attenuated the increase in ISC following pretreatment. In addition, disruption of endogenous H_2_S synthesis impaired embryo implantation. The findings indicate that H_2_S suppresses the secretion of transepithelial anions in mouse endometrial epithelium by blocking CFTR during early-stage pregnancy, thereby regulating the uterine fluid volume to prepare for embryo implantation.

### 5.4. H_2_S Promotes Postpartum Myometrial Recovery

During pregnancy and childbirth, the uterus undergoes molecular and functional changes that affect the regulation of H_2_S. It was shown that L-Cys decreased the extent of natural constrictions in non-laboring or laboring myometrial tissues in a dose-dependent manner, according to You et al. [95]. Elevated levels of L-Cys heightened the frequency of natural uterine constrictions and triggered sustained constrictions. GLB pretreatment attenuated the suppressive impact of L-Cys on the strength of natural contractions in myometrium strips, indicating that L-Cys hinders uterine muscle constrictions through the stimulation of K_ATP_ channels. Compared with non-laboring myometrial tissues, laboring myometrial tissues had decreased CBS and CSE levels. In addition, the amplitude of spontaneous contractions and baseline muscle tension were less affected by L-Cys in laboring myometrial tissues. These findings suggest that decreased levels of H_2_S contribute to the transition of the uterus from quiescence to contraction after parturition. Therefore, H_2_S can be used to prevent infection-related preterm births and treat endometriosis. Overall, H_2_S has a beneficial impact on controlling biological functions such as angiogenesis in the proliferative phase of the female menstrual cycle and pregnancy; the formation of a favorable uterine fluid microenvironment for embryo implantation; and the recovery of myometrium after delivery, which is important for maintaining the integrity of uterine structure and function.

### 5.5. H_2_S Improves Pre-Eclampsia

Placental abnormalities can lead to severe complications like pre-eclampsia and restricted fetal growth. The regulatory effects of H_2_S on the placenta are involved in the pathogenesis of pre-eclampsia. Sarno et al. [102] indicated that the generation of H_2_S in postpartum placental models of early-onset and late-onset pre-eclampsia was comparable to that in the placenta of pregnant women without health issues. Following the addition of L-Cys, the late-onset pre-eclampsia group exhibited increased H_2_S production compared with the normal pregnancy and early-onset pre-eclampsia groups. Nevertheless, research has indicated that the mRNA levels of CBS are lower in placental models of early-onset pre-eclampsia compared with placentas from healthy pregnant individuals, while CSE expression remains unchanged. The decreased mRNA levels of CBS result in reduced H_2_S production [58]. The levels of CSE/H_2_S are lower in the plasma and placental tissues of pregnant women with pre-eclampsia than in those of healthy pregnant women. Notably, the decreased expression of H_2_S and CSE leads to maternal hypertension and placental abnormalities [60]. Although these findings are contradictory, they suggest the potential involvement of the L-Cys/H_2_S pathway in the pathogenesis of pre-eclampsia. One of the major factors involved in the development of pre-eclampsia is the increased expression of soluble FMS-like tyrosine kinase 1 (sFlt-1) and soluble endocrine hormone (sEng) in the placenta [149]. Studies have shown that CSE knockdown increases the release of sFlt-1 and sEng from human umbilical vein endothelial cells, whereas its overexpression inhibits the release of the two factors. Moreover, treatment with GYY4137 can also inhibit sFlt-1 and sEng at the circulating level [60,150]. Increased sFlt-1 expression has been strongly associated with increased levels of metalloproteinase 10 (ADAM10). ADAM10 levels are higher in placenta samples from women with pre-eclampsia than in those from healthy women and are negatively correlated with the levels of CBS and CSE. Silencing of ADAM10 leads to decreased sFlt-1 release, while administration of NaHS and L-Cys effectively suppresses ADAM10 expression [103]. Hu et al. [59] showed that when syncytiotrophoblasts obtained from the placentas of both healthy women and women with pre-eclampsia were exposed to NaHS and L-Cys in culture, the protein expression of sFlt-1 was decreased, the half-life of sFlt-1 mRNA was shortened, and the expression of miR-133b targeting sFlt-1 was increased. These results suggest that H_2_S delays the development of pre-eclampsia by suppressing the release of sFlt-1. The progression of pre-eclampsia is driven by TLR4-mediated sympathetic hyperactivity caused by inflammation of the rostral ventrolateral medulla oblongata (RVLM). Research [104] indicated elevated plasma levels of inflammatory markers and norepinephrine, along with decreased levels of H_2_S, in pre-eclampsia patients compared with healthy pregnant individuals. Similarly, compared with normal rats, rats with deoxycorticosterone acetate-induced pre-eclampsia exhibited higher renal sympathetic activity, higher plasma norepinephrine levels, and lower H_2_S levels in RVLM. Injection of the TLR4 agonist LPS induced microglia-mediated inflammation in RVLM, increased the sympathetic tone, and aggravated pre-eclampsia-like symptoms in pregnant rats. However, administration of NaHS alleviated these manifestations in both rats with PE and pregnant rats treated with LPS. These findings suggest that H_2_S reduces the severity of pre-eclampsia by inhibiting TLR4 and attenuating inflammatory responses in RVLM, providing a new target for the treatment of pre-eclampsia. Placental health relies on endogenous H_2_S, and decreased H_2_S levels in vivo may contribute to the development and progression of pre-eclampsia.

**Figure 7:**
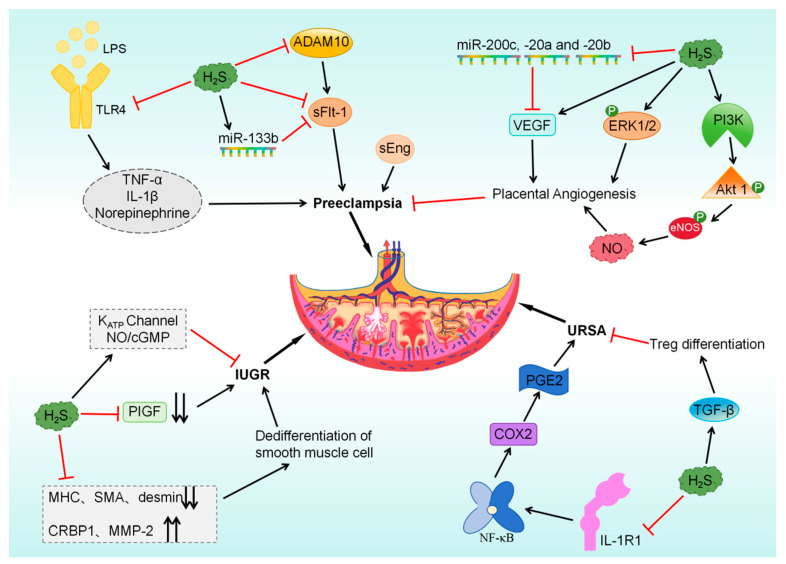
Regulatory mechanism of H_2_S in pre-eclampsia, URSA, and IUGR. URSA: unexplained recurrent spontaneous abortion; IUGR: intrauterine growth restriction.

Pre-eclampsia is closely associated with an imbalance of angiogenic growth factors and is characterized by an antiangiogenic state. Consequently, the pro-angiogenic effects of H_2_S on the placenta may contribute to the alleviation of pre-eclampsia. H_2_S-miRNA has been reported to regulate vascular endothelial growth factor (VEGF) in the placenta and promote placental angiogenesis, thereby ameliorating pre-eclampsia [105]. CBS, CSE, VEGF, miR-200c, miR-20a, and miR-20b are downregulated in placentas during pre-eclampsia. In a study, treatment of explants and trophoblasts isolated from healthy placentas with H_2_S donors and L-Cys elevated the protein expression of VEGF, prolonged the half-life of VEGF mRNA, and decreased the expression of miR-200c, miR-20a, and miR-20b. Unlike the mimics and inhibitors of miR-200c, those of miR-20a or miR-20b affect VEGF expression at the protein level but not at the mRNA level [105]. Chen et al. [106] showed that HTR-8/SVneo cells from villous trophoblasts increased the migratory capacity of sheep placental arterial endothelial cells (oFPAECs), while the use of CBS inhibitors reduced this impact. In oFPAECs, the effects of H_2_S donors on angiogenesis were comparable to those of VEGF; however, the rapid activation of eNOS, Akt1, and ERK1/2 upon H_2_S donor phosphorylation was slightly weaker than that upon VEGF phosphorylation. ERK1/2, PI3K/Akt1, and eNOS/NO are key signaling pathways that mediate angiogenesis. When activated by H_2_S donors, specific PI3K inhibitors can block the phosphorylation of Akt1 and eNOS without affecting ERK1/2. Similarly, ERK1/2 inhibitors do not affect the phosphorylation of Akt1 and eNOS. These findings suggest that H_2_S derived from trophoblasts stimulates placental angiogenesis through phosphorylation of the PI3K/Akt1/eNOS and ERK1/2 pathways in endothelial cells. Decreased expression of placental growth factor (PIGF) indicates impaired placental angiogenesis. In vitro studies have shown that PAG decreases the production of PIGF and hinders the invasion of trophoblasts in human placental explants in the initial stages of pregnancy. PAG can lead to high blood pressure and liver impairment in pregnant mice, promote the development of abnormal labyrinth vessels in the placenta, and cause fetal development restriction (Figure 7). However, treatment with GYY4137 counteracts the effects of PAG [60,150]. Consequently, dysfunction of the CSE/H_2_S system may lead to placental abnormalities in pre-eclampsia and affect normal fetal development.

### 5.6. H_2_S Improves Fetal Growth Restriction

Impaired blood flow in the umbilical artery and elevated placental vascular resistance are frequently linked to intrauterine growth restriction (IUGR) [151]. In addition, pre-eclampsia limits fetal growth and development in utero. It was demonstrated that the expression of CSE was notably lessened and that of miR-21, a negative regulator of CSE expression, was markedly elevated in IUGR and pre-eclampsia with abnormal Doppler umbilical artery waveform (PE-AD) placentas than in healthy placentas [61]. The same expression patterns were observed in villus explants subjected to hypoxia-reoxygenation damage and placental stem villus arteries (SVAs) [61,108]. The smooth muscle phenotype of SVAs is in a state of dedifferentiation, with decreased expression of SMC differentiation markers such as myosin heavy chain, smooth muscle actin, and desmin and increased expression of dedifferentiation markers such as retinol-binding protein 1 and MMP-2. Notably, under normoxic conditions, PAG results in a dedifferentiated state of SMCs in SVA tissues ex vivo, whereas treatment with the H_2_S donor diallyl trisulfide reverses the effects of PAG. Lu et al. [108] found that neonatal birth weight had a positive correlation with the expression of CSE and SMC differentiation markers but a negative correlation with SMC dedifferentiation markers in placental tissues. Consistently, placental SVAs at IUGR had a smaller lumen diameter and unchanged wall thickness than at term delivery or preterm labor and were also associated with the loss of end-diastolic flow. These findings suggest that IUGR is involved in the vascular remodeling of SVAs induced by abnormal CSE/H_2_S signaling. NaHS relaxes blood vessels in normal placentas pre-constricted with the thromboxane A2 mimetic U46619 in a dose-dependent manner (Figure 7). These relaxant effects of NaHS can be reversed by GLB and L-NAME [61]. Therefore, H_2_S donors may reduce placental blood flow obstruction by preventing the dedifferentiation of SMCs and dilating blood vessels through the K_ATP_ channel and NO/cGMP pathway, which may enable normal fetal growth and development.

### 5.7. H_2_S Alleviates Recurrent Spontaneous Abortion

Immune homeostasis is often dysregulated in unexplained recurrent spontaneous abortion (URSA) [152]. H_2_S can regulate immune homeostasis by promoting the differentiation of regulatory T cells (Tregs), thus helping the fetus to escape the attack of the maternal immune system on paternal antigens [153,154]. Wang et al. [109] showed that treatment of decidual tissues from pregnant mice with AOAA or PAG significantly reduced the production of TGFβ, a crucial factor for the growth, specialization, and cell identification abilities of Treg cells [155]. However, treatment with GYY4137 or NaHS effectively restored the expression of TGFβ. At the time of embryo implantation, destruction of the luminal epithelium initiates the inflammatory response, and the maternal immune system is reprogrammed from the T-helper 1 (Th1) state to the Th2 state to ensure immune tolerance during pregnancy [156,157]. In a study, the decidua of resorbed fetuses exhibited increased levels of Th1 cytokines (IFN-γ and TNF-α) and decreased levels of Th2 cytokines (IL-4 and IL-6), validating the relationship between immune homeostasis and URSA. CBS/H_2_S in HTR8/SVneo cells could potentially suppress NF-κB signaling pathway activation by decreasing IL-1R1 levels, leading to decreased expression of inflammatory response factors like COX2 and reduced PGE2 secretion [109]. Abnormal expression of PGE2 and COX2 is the cause of URSA [158,159]. In early human pregnancy, H_2_S regulates three immune tolerance-related proteins, namely, human chorionic gonadotropin, diamide-2,3-dioxygenase [160,161,162,163,164], and thymic stromal lymphopoietin [165], which maintains the Th2 state in the decidua. Inadequate trophoblast invasion may lead to pregnancy loss in the early stages [166]. CBS expression is decreased in placental villous cytotrophoblasts in URSA compared with normal early pregnancy, while CSE expression remains unchanged. Pregnant mice lacking CBS or treated with AOAA show a notable rise in embryo resorption rate and percentage of embryo loss, both of which can be decreased effectively with GYY4137 or NaHS. Exogenous administration of H_2_S enhances the migratory and invasive abilities of HTR8/SVneo and JEG3 cells (human placental trophoblasts), along with increasing the levels of MMP-2 and VEGF proteins [109] (Figure 7). Overall, placental CBS/H_2_S signaling plays a crucial role in early pregnancy by regulating immune tolerance and trophoblast invasion.

### 5.8. H_2_S Improves Placental Oxidative Damage

Various placental complications are associated with smoking during pregnancy or exposure to cigarette smoke during pregnancy [167,168]. Zhao et al. [110] showed that exposure of pregnant rats to cigarette smoke on days 7–20 of gestation increased the levels of 8-OHdG, DNA oxidative damage markers, and MDA and aberrantly decreased the levels of Nrf2 in the placenta, suggesting oxidative damage in the placenta. However, the administration of NaHS significantly counteracted these changes. Furthermore, it successfully decreased the placental redox imbalance caused by cigarette smoke by reinstating the overall antioxidant capacity, elevating the GSH/GSSG ratio, and boosting the functions of SOD, catalase, and glutathione peroxidase to prevent ROS generation. The results indicate that H_2_S decreases the generation of ROS caused by cigarette smoke exposure in pregnant women by activating the Nrf2 pathway, ultimately reducing oxidative harm to the placenta.

The placenta is crucial in the pathogenesis of gestational diabetes mellitus (GDM) [169]. In GDM, deficiency of H_2_S-producing enzymes in the placenta results in excessive activation of the NLRP3 inflammasome, an important inflammatory cytokine involved in the initiation of maternal insulin resistance [111]. Therefore, H_2_S may be involved in the pathogenesis of GDM by controlling NLPR3 inflammasome activation in the placenta. Altogether, H_2_S plays an essential role in promoting normal placental angiogenesis and blood flow, maintaining placental immune homeostasis in early pregnancy, and facilitating the growth and development of embryos after implantation. Dysfunction of the H_2_S system is involved in pre-eclampsia, URSA, IUGR, and other placenta-related diseases caused by various factors, whereas H_2_S supplementation plays an effective therapeutic role in these diseases.

### 5.9. H_2_S Promotes Ovulation

Elevated homocysteine levels may affect oocyte quality or interfere with ovulation. CBS is the main enzyme involved in homocysteine metabolism and is widely distributed in the ovary. An impairment of CBS activity can lead to hyperhomocysteinemia [101]. Liang et al. [112] found that CBS levels were decreased in mouse ovarian granulosa cells, whereas homocysteine and methionine levels were notably increased in the follicular fluid. Furthermore, the rate of in vitro oocyte maturation was significantly reduced in the CBS-inhibited granulosa cell medium cultured oocyte model. CBS-knockout females have been shown to develop fewer follicles than wild-type female rats [101]. The results indicate that CBS is responsible for controlling oocyte maturation through the maintenance of homocysteine levels in the oocyte environment. Before ovulation, the expression of CSE in granulosa cells is increased by the upregulation of luteinizing hormone (LH), leading to higher levels of mRNA and protein. Administration of NaHS can enhance the mRNA levels of proteins related to cumulus expansion and follicle rupture, including amphiregulin, epiregulin, and plasminogen activator, in mouse granulosa cells, thus facilitating ovulation. However, CSE inhibitors can block ovulation [170]. Altogether, H_2_S has positive effects on germ cell development in the ovary.

### 5.10. H_2_S Regulates Oviductal Transport

The fallopian tube provides a favorable microenvironment for the final maturation of gametes, fertilization, and the primary stage of embryonic development [171]. However, if the embryo is retained in the fallopian tube, it can lead to an ectopic pregnancy. Typically, the fallopian tube’s smooth muscle contracts regularly like a sphincter in normal circumstances. Ning et al. [26] showed that administration of NaHS or L-Cys resulted in the relaxation of the smooth muscle in the fallopian tube in a manner that depended on the dosage. Additionally, there was a notable rise in the levels of CBS and CSE in the epithelial cells of the fallopian tube in cases of ectopic pregnancy. Treatment with an H_2_S donor and a CBS activator impaired embryonic trafficking and induced asynchronous development in mice. In addition, the effects of a CBS inhibitor were reversed after administration of NaHS or GYY4137. These findings suggest that changes in H_2_S signaling in the oviduct of pregnant mice delay embryo transport and early embryonic development, resulting in the failure of timely embryo implantation in the uterus. The normal transmission of H_2_S signals is essential for the oviductal transport of embryos.

### 5.11. H_2_S Modulates the Female Sexual Response

In addition, H_2_S plays an important role in female sexual responses. Srilatha et al. [16] showed that NaHS significantly relaxed rabbit vaginal smooth muscle strips in a way that depended on the concentration; however, these relaxation effects were reversed after treatment with MDL-12,330a (an adenylate cyclase inhibitor), L-NAME, ODQ, or GLB. The results indicate that the relaxation properties of H_2_S on rabbit vaginal smooth muscle are mainly related to cAMP, NO/cGMP, and K_ATP_ channels. Sun et al. [65] showed that treatment with NaHS increased vaginal lubrication, which was related to the H_2_S-induced increase in the concentrations of K^+^ and Cl^−^ ions in rat vaginal fluid. Furthermore, treatment with NaHS initially decreased ISC but subsequently increased it in isolated rat vaginal tissues. The decrease in ISC was sensitive to K_ATP_ channel inhibitors, whereas the increase in ISC relied on Cl^-^ ions and could be attenuated by CFTR inhibitors. Both inhibitors prevented NaHS-induced vaginal lubrication in vivo. Given that CSE is the main enzyme expressed in the vaginal epithelium, the CSE/H_2_S pathway is crucial in regulating the secretion of vaginal fluid.

Overall, the pathway for producing H_2_S is crucial for the maturation of oocytes and ovulation, as well as the transportation and growth of early tubal embryos and the regulation of vaginal smooth muscle relaxation and vaginal fluid secretion. On the contrary, abnormal H_2_S signaling can inhibit ovulation, delay embryonic development, and lead to vaginal dryness. These pathological conditions can be treated by the exogenous administration of H_2_S donors.

## 6. Conclusions and Discussion

In conclusion, H_2_S regulates both male and female reproductive systems and participates in physiological processes such as penile erection, testicular reproduction, spontaneous contraction of the vas deferens, ejaculation, ovulation, pregnancy, and postpartum recovery, thus maintaining the normal structure and function of reproductive organs. Abnormal H_2_S signaling may lead to andrological conditions such as ED, male infertility, premature ejaculation, and spermatorrhea, as well as obstetric conditions such as embryo implantation failure, recurrent miscarriages, fetal growth restriction, pre-eclampsia, and premature delivery. The mechanisms through which H_2_S promotes penile erection remain unclear. H_2_S participates in muscle relaxation through activation of BKCa and Kv channels, inhibition of the RhoA/ROCK signaling pathway, and upregulation of cGMP in an NO-independent manner. Ghasemi et al. [50] found that H_2_S relaxed the corpus cavernosum at pharmacological concentrations while inhibiting the relaxation of NO at physiologically relevant concentrations. Studies reporting similar findings are limited; therefore, further investigation is necessary. H2S has been shown to alleviate ED by inhibiting superoxide production in cavernosum and vascular SMCs [68]. Superoxide produced by NADPH oxidase (NOX) can reduce the bioavailability of NO by reacting with it to form reactive nitrogen species [172,173,174,175], increase cGMP metabolism by upregulating PDE5, and activate ROCK [176], eventually impairing erectile function. NO can inhibit the activity and expression of NOX through the cGPM-PKG pathway [177,178,179]; consequently, diseases or conditions characterized by impaired endothelial structure, such as diabetes, are predisposed to ED. The inhibitory effects of H_2_S on superoxide production are complex. H_2_S not only inhibits the activity and expression of NOX through the cAMP-PKA and cGMP-PKG pathways [180,181] but also regulates the expression of Nrf2 and its downstream anti-oxidative stress proteins, including SOD, NAD(P)H quinone oxidoreductase, and HO-1, through S-sulfhydration of Keap1, consequently suppressing superoxide production [182]. In addition, S-sulfhydration of H_2_S is involved in the activation of K_ATP_ channels [183] and BKCa channels [24] and the reduction of PDE5A dimerization [184]. The mechanisms through which H_2_S regulates female reproductive organs are complex and diverse but have not been investigated comprehensively. Relaxation of uterine vessels, placental vessels, and vaginal smooth muscle relies on K_ATP_ channels, which are associated with NO/cGMP signaling. In different environments, H_2_S can regulate inflammation and cell proliferation by selectively activating or inhibiting ERK1/2 and NF-κB, alleviating pre-eclampsia and preventing infection-related preterm birth. With in-depth research on H_2_S, various H_2_S-based therapeutic strategies and H_2_S-releasing drugs, including ZnS@TA [123], ACS6 [20], HS-NSAID [121], and H_2_SDox [122], have been developed for the treatment of reproductive system diseases. However, studies investigating the biological role of H_2_S in the reproductive system are fewer than those focusing on other human organ systems; moreover, a systematic description is lacking. Hence, further research is needed to explore the impact of H_2_S on the reproductive system in order to enhance the efficacy of treatments for reproductive system disorders.

## Figures and Tables

**Table 1:** Effects of H_2_S on the reproductive system.

Organ	Action	Mechanisms	Models	References
Penis	Promotion of erection	Activation of RhoA/ROCK pathway and K_ATP_ channel	HCC strips from transsexual procedures (*n* = 6)	[17]
		Activation of RhoA/ROCK pathway	Bilateral cavernous nerve injury rats (*n* = 18)	[19]
		Dependent on cAMP or cGMP	Rabbit CC strips (*n* = 5)	[47]
		Activation of BKCa channel	Anesthetized rat	[67]
		Increase eNOS expression	L-NAME-induced hypertensive rats (*n* = 40)	[68]
		Activation of sGC/cGMP pathway	HCC strips of men with ED (*n* = 50)	[81]
		Increase the levels of NO and HO activity	Diabetic rats (*n* = 90)	[82]
		Activation of NO/sGC/cGMP pathway	CSE^−/−^ mice	[83]
		Activation of BKCa channel and Kv channel	Rat CC strips	[84]
		Activation of NO/sGC/cGMP pathway and K_ATP_ channel	STZ-diabetic rats (*n* = 10 or 12)	[85]
		Activation of RhoA/ROCK pathway	Mouse CC strips	[86]
	Improve vascular injury of CC	Inhibition of TGF-β1/Smad/CTGF pathway	STZ-diabetic rats	[77]
Prostate	Inhibition of CRPC	S-sulfhydrylation of AR	CSE knock-out and overexpression LNCaP; LNCaP-B	[54]
	Promotion of NE differentiation	Enhance the activity of Cav3.2	LNCaP	[87]
Testis	Alleviate the apoptosis of testicular germ cells	Increase SOD activity and reduce Bax/Bcl-2 ratio	Heat exposure treated mice	[55]
	Reduce sperm motility	Activation of AMPK/Akt related pathways	Boar sperm;NH_4_Cl and/or Na_2_S treated mice	[88]
		Activation of Keap1/Nrf2 signaling pathway	GC-2spd(ts) cells derived from mouse spermatocytes	[89]
		Reduction of iNOS, TNF-α, and Apaf-1	Testicular torsion-induced I/R injury rat model (*n* = 38)	[90]
	Alleviate testosterone synthesis	Sulfhydrylation of PDE4A/8A and activation of cAMP/PKA pathway	LPS+ induced testosterone synthesis impairment model of mouse Leydig tumour cells	[91]
	Increase sperm motility	Activation of CBS/H_2_S pathway	LPS− and diabetes-induced sperm dysfunction mice model;CBS^+/−^ mice model	[92]
		Scavenge ROS	Fe^2+^/ascorbate treated boar sperm	[93]
Epididymis	Maintain quiescence of epididymal sperm	Activation of K_ATP_ channel and BKCa channel	Cauda epididymal epithelium cells	[56]
	Alleviate varicocele-induced epididymis injury	Activation of PI3K/Akt pathway	Experimental varicocele rat model (*n* = 60)	[94]
Vas deferens	Regulation of VD spontaneous contraction	Activation of L-Cys/H_2_S pathway	Human VDs from monorchidism surgery (*n* = 3); rat VDs (*n* = 20); mice VDs (*n* = 11)	[23]
		Activation of BKCa channel	Rat VDs	[24]
Uterus	Abnormal contraction of uterus	Activation of L-Cys/CSE/H_2_S	L-Cys and sildenafil treated uterus collected from CSE^−/−^ mice (*n* = 10).	[74]
		Activation of K_ATP_ channel	L-Cys treated Human myometrium (*n* = 6).	[95]
	Premature delivery	Inhibition of TLR4/NF-κB signaling pathway	LPS-induced C57BL/6 mice (*n* = 8).	[96]
			IL-1β treated USMCs obtained from TL and TNL myometrium (*n* = 12).	
		Inhibition of ERK1/2 and NF-κB signaling pathway	LPS-induced BALB/c mice (*n* = 10).	[97]
	Promote uterine angiogenesis	Improve the expression of CBS	UA collected from female uterus (NP, *n* = 20; P, *n* = 10);	[62]
			Phenylephrine-treated UA collected from SD mice (*n* = 8–10).	
			E_2_-treated HESCs isolated from human endometrial	[64]
			(POM, *n* = 9; pPRM, *n* = 13; sPRM, *n* = 4; Preg, *n* = 13).	
			ERT-treated UA isolated from ovx NP ewes (*n* = 3–5).	[98]
			E_2_-treated UASMCs isolated from late pregnant ewes (*n* = 3–5).	[99]
	Endometriosis	Activation of NF-κB	HESCs isolated from human endometrium (*n* = 21); Allogeneic endometrial fragment transplantation induced BABL/C mice (*n* = 6).	[100]
	Decreased uterine fertility	Replenish the CBS	Ovary transplantation treated CBS^−/−^ mice.	[101]
Placenta	Preeclampsia	Activation of CSE/H_2_S pathway	Placenta and plasma collected from women with PE and normotensive pregnant (*n* = 14); PAG-treated C57BL/6 pregnant mice.	[60]
		Activation of K_ATP_ channel	IUGR and PE placenta collected from female (*n* = 6 and 13).	[61]
		Promotion of NO synthesis	Hypoxia-treated placental explants; bicarbonate buffer treated healthy placenta (*n* = 8).	
		Activation of L-cys/H_2_S pathway	Placenta collected from women with PE (*n* = 10); L-Cys treated SHR.	[102]
		Inhibition of SFlt-1 expression	Placenta obtained from woman with PE (*n* = 19);	[59]
			NaHS and L-Cys treated syncytiotrophoblasts.	
			Placenta collected from women with PE (*n* = 18);	[103]
			NaHS and L-Cys treated cytotrophoblasts	
		Inhibition of TLR4 expression	Plasma collected from women with PE (*n* = 30);	[104]
			DOCA-salt and LPS treated SD pregnant rats (*n* = 6).	
		Activation of VEGF expression	Placenta obtained from women with PE (*n* = 19); NaHS and L-Cys treated explants and trophoblasts isolated from healthy placentas.	[105]
		Activation of PI3K/Akt1/eNOS and ERK1/2 signaling pathway	NaHS-treated oFPAECs isolated from sheep placental arteries.	[106]
			BCA and CHH treated HTR-8/SVneo.	
	Promotion of embryo implantation	Inhibition of CFTR expression	AOAA or PAG treated pregnant KM rat (*n* = 3);	[107]
			NaHS-treated MEECs isolated from KM female rat.	
	Intrauterine fetal growth restriction	Activation of CSE/H_2_S pathway	HR-induced SVA collected from human IUGR placentas (*n* = 34).	[108]
	Recurrent spontaneous abortion	Activation of CBS/H_2_S pathway	AOAA and PAG treated C57BL/6 mice; GYY4137 and NaHS treated CBS^+/−^♀ mice;	[109]
			NaHS or L-Cys treated HTR8/SVneo and JEG3 cells.	
	Promotion placental development	Elevation of Nrf2 expression	Cigarette smoke induced SD pregnancy rats.	[110]
	Gestational diabetes	Inhibition of NLPR3	Placenta collected from pregnant women with GDM (*n* = 16);	[111]
			NaHS or L-Cys treated HTR-8/SVneo.	
Ovary	Promotion of ovulation	Elevation of CBS and CSE expression	PMSG-treated granulosa cells isolated from ICR mice ovary.	[112]
Fallopian tube	Promotion of tubal transport	Activation of H_2_S signaling pathway	NaHS or L-Cys treated fallopian tubes collected from female (*n* = 7 or 5);	[26]
			AOAA-treated C57BL/6J mice and BALB/c mice (*n* = 22).	
Vagina	Sexual response	Activation of CAMP, NO-cGMP, K_ATP_ signaling pathway	NaHS-treated Vaginal isolated from white rabbits (*n* = 12).	[16]
		Activation of CSE/H_2_S pathway	NaHS-treated SD rats (*n* = 10); NaHS-treated VK2/E6E7.	[65]

**Abbreviation:** L-Cys: L-cysteine, LPS: lipopolysaccharide, USMCs: uterine smooth muscle cells, TL: term in labour, TNL: term no labour, HESCs: human endometrial stromal cells, POM: postmenopausal, pPRM: proliferative premenopausal, sPRM: secretory premenopausal, Preg: pregnant, ERT: estrogen replacement therapy, UA: uterine artery, OVX: ovariectomized, NP: nonpregnant, P: pregnant, E_2_: estradiol-17β, UASMC: uterine artery smooth muscle cell, SD: Sprague Dawley, PE: preeclampsia, SHR: sponta hypertensive rats, PAG: DL-propargylglycine, HUVECs: human umbilical vein endothelial cells, IUGR: intrauterine fetal growth restriction, oFPAECs: ovine fetoplacental artery endothelial cells, BCA: β-cyano-L-alanine, CHH: carboxymethyl hydroxylamine hemihydrochloride, DOCA: desoxycorticosterone acetate, AOAA: amino-oxyacetic acid, KM: kunming, MEECs: murine primary uterine endometrial epithelial cells, HR: hypoxia reoxygenation, SVA: stem villus arterie, WT; wild-type, GDMz; gestational diabetes mellitus, PMSGzzz; pregnant mare’s serum gonadotropin, Akt: protein kinase B, AMPK: adenosine 5′-monophosphate AMP-activated protein kinase, Apaf-1: apoptosis protease activating factor-1, CRPC: castration-resistant prostate cancer, BKCa: large-conductance Ca^2+^-activated K^+^, cAMP: cyclic adenosine monophosphate, cGMP: cyclic guanosine monophosphate, CTGF: connective tissue growth factor, CC: corpus cavernosum, ED: erectile dysfunction, eNOS: endothelial NOS, ERK1/2: extracellular signal-regulated kinase 1/2, HCC: human corpus cavernosum, HO: heme oxygenase, iNOS: inducible NOS, Keap1: Kelch-like ECH-associated protein 1, L-NAME: nω-nitro-L-arginine, NE: neuroendocrine, NF-κB: nuclear factor-κB, PCa: prostate cancer, ROS: oxygen species, Nrf2: nuclear factor erythroid 2-related factor 2, PI3K: phosphatidylinositol 3′-OH kinase, ROCK: Rho-kinase, sGC: soluble guanylate cyclase, SOD: superoxide dismutase, STZ: streptozotocin, TGF-β1: transforming growth factor-β1, TNF-α: tumor necrosis factor-α, VD: vas deferens.

## References

[1] Miyaso H., Ogawa Y., Itoh M. (2022). Microenvironment for spermatogenesis and sperm maturation. Histochem. Cell Biol..

[2] Magro-Lopez E., Muñoz-Fernández M.Á. (2021). The role of bmp signaling in female reproductive system development and function. Int. J. Mol. Sci..

[3] Farsimadan M., Motamedifar M. (2020). Bacterial infection of the male reproductive system causing infertility. J. Reprod. Immunol..

[4] Han X., Huang Q. (2021). Environmental pollutants exposure and male reproductive toxicity: The role of epigenetic modifications. Toxicology.

[5] Fozooni R., Shirazi M.R.J., Saedi S., Jahromi B.N., Khoradmehr A., Anvari M., Rahmanifar F., Khodabandeh Z., Tamadon A. (2021). Male subfertility effects of sub-chronic ethanol exposure during stress in a rat model. Alcohol.

[6] He L., Gong H., You S., Zhang C., Zhong C., Li L. (2021). Mirna-138-5p suppresses cigarette smoke-induced apoptosis in testicular cells by targeting caspase-3 through the bcl-2 signaling pathway. J. Biochem. Mol. Toxicol..

[7] Chacra L.A., Benatmane A., Iwaza R., Ly C., Alibar S., Armstrong N., Mediannikov O., Bretelle F., Fenollar F. (2023). Culturomics reveals a hidden world of vaginal microbiota with the isolation of 206 bacteria from a single vaginal sample. Arch. Microbiol..

[8] Wu X., Tian Y., Zhu H., Xu P., Zhang J., Hu Y., Ji X., Yan R., Yue H., Sang N. (2023). Invisible Hand behind Female Reproductive Disorders: Bisphenols, Recent Evidence and Future Perspectives. Toxics.

[9] Yang G., Wang R. (2015). H_2_S and blood vessels: An overview. Handb. Exp. Pharmacol..

[10] Cirino G., Szabo C., Papapetropoulos A. (2023). Physiological roles of hydrogen sulfide in mammalian cells, tissues, and organs. Physiol. Rev..

[11] Zhu X.-Y., Gu H., Ni X. (2011). Hydrogen sulfide in the endocrine and reproductive systems. Expert Rev. Clin. Pharmacol..

[12] Bianca R.D.d.V., Fusco F., Mirone V., Cirino G., Sorrentino R. (2017). The Role of the Hydrogen Sulfide Pathway in Male and Female Urogenital System in Health and Disease. Antioxid. Redox Signal.

[13] Wang R., Tang C. (2022). Hydrogen Sulfide Biomedical Research in China—20 Years of Hindsight. Antioxidants.

[14] Kadlec M., Ros-Santaella J.L., Pintus E. (2020). The Roles of NO and H_2_S in Sperm Biology: Recent Advances and New Perspectives. Int. J. Mol. Sci..

[15] Liang R., Yu W.-D., Du J.-B., Yang L.-J., Shang M., Guo J.-Z. (2006). Localization of cystathionine beta synthase in mice ovaries and its expression profile during follicular development. Chin. Med. J..

[16] Srilatha B., Hu L., Adaikan G.P., Moore P.K. (2009). Initial Characterization of Hydrogen Sulfide Effects in Female Sexual Function. J. Sex. Med..

[17] d’Emmanuele di Villa Bianca R., Sorrentino R., Maffia P., Mirone V., Imbimbo C., Fusco F., De Palma R., Ignarro L.J., Cirino G. (2009). Hydrogen sulfide as a mediator of human corpus cavernosum smooth-muscle relaxation. Proc. Natl. Acad. Sci. USA.

[18] Srilatha B., Adaikan P.G., Moore P.K. (2006). Possible role for the novel gasotransmitter hydrogen sulphide in erectile dysfunction—A pilot study. Eur. J. Pharmacol..

[19] Zeng Q., He S., Chen F., Wang L., Zhong L., Hui J., Ding W., Fan J., Zhang H., Wei A.-Y. (2021). Administration of h(2)s improves erectile dysfunction by inhibiting phenotypic modulation of corpus cavernosum smooth muscle in bilateral cavernous nerve injury rats. Nitric Oxide Biol. Chem..

[20] Shukla N., Rossoni G., Hotston M., Sparatore A., Del Soldato P., Tazzari V., Persad R., Angelini G.D., Jeremy J.Y. (2009). Effect of hydrogen sulphide-donating sildenafil (acs6) on erectile function and oxidative stress in rabbit isolated corpus cavernosum and in hypertensive rats. BJU Int..

[21] Sugiura Y., Kashiba M., Maruyama K., Hoshikawa K., Sasaki R., Saito K., Kimura H., Goda N., Suematsu M. (2005). Cadmium Exposure Alters Metabolomics of Sulfur-Containing Amino Acids in Rat Testes. Antioxid. Redox Signal..

[22] Ová H.I., Moravec J.Í., Tiavnická M., Havránková J.I., Monsef L., Ek P.H., Ová Á.P., Almanová T., Fenclová T., Petr J. (2022). Evidence of endogenously produced hydrogen sulfide (h(2)s) and persulfidation in male reproduction. Sci. Rep..

[23] Li J., Li Y., Du Y., Mou K., Sun H., Zang Y., Liu C. (2011). Endogenous hydrogen sulfide as a mediator of vas deferens smooth muscle relaxation. Fertil. Steril..

[24] Li Y., Zang Y., Fu S., Zhang H., Gao L., Li J. (2012). H_2_S Relaxes Vas Deferens Smooth Muscle by Modulating the Large Conductance Ca^2+^-Activated K^+^ (BKCa) Channels via a Redox Mechanism. J. Sex. Med..

[25] Sidhu R., Singh M., Samir G., Carson R.J. (2001). L-cysteine and sodium hydrosulphide inhibit spontaneous contractility in isolated pregnant rat uterine strips in vitro. Pharmacol. Toxicol..

[26] Ning N., Zhu J., Du Y., Gao X., Liu C., Li J. (2014). Dysregulation of hydrogen sulphide metabolism impairs oviductal transport of embryos. Nat. Commun..

[27] Nuño-Ayala M., Guillén N., Arnal C., Lou-Bonafonte J.M., de Martino A., García-De-Jalón J.-A., Gascón S., Osaba L., Osada J., Navarro M.-A. (2012). Cystathionine β-synthase deficiency causes infertility by impairing decidualization and gene expression networks in uterus implantation sites. Physiol. Genom..

[28] Wang R. (2012). Physiological Implications of Hydrogen Sulfide: A Whiff Exploration That Blossomed. Physiol. Rev..

[29] Kolluru G.K., Shackelford R.E., Shen X., Dominic P., Kevil C.G. (2023). Sulfide regulation of cardiovascular function in health and disease. Nat. Rev. Cardiol..

[30] Singh S., Padovani D., Leslie R.A., Chiku T., Banerjee R. (2009). Relative contributions of cystathionine beta-synthase and gam-ma-cystathionase to H_2_S biogenesis via alternative trans-sulfuration reactions. J. Biol. Chem..

[31] Munteanu C., Rotariu M., Turnea M., Dogaru G., Popescu C., Spînu A., Andone I., Postoiu R., Ionescu E.V., Oprea C. (2022). Recent Advances in Molecular Research on Hydrogen Sulfide (H_2_S) Role in Diabetes Mellitus (DM)—A Systematic Review. Int. J. Mol. Sci..

[32] Li L., Moore P.K. (2008). Putative biological roles of hydrogen sulfide in health and disease: A breath of not so fresh air?. Trends Pharmacol. Sci..

[33] Szabó C. (2007). Hydrogen sulphide and its therapeutic potential. Nat. Rev. Drug Discov..

[34] Liu J., Mesfin F.M., Hunter C.E., Olson K.R., Shelley W.C., Brokaw J.P., Manohar K., Markel T.A. (2022). Recent Development of the Molecular and Cellular Mechanisms of Hydrogen Sulfide Gasotransmitter. Antioxidants.

[35] Lv B., Chen S., Tang C., Jin H., Du J., Huang Y. (2021). Hydrogen sulfide and vascular regulation—An update. J. Adv. Res..

[36] Zou S., Shimizu T., Shimizu S., Higashi Y., Nakamura K., Ono H., Aratake T., Saito M. (2018). Possible role of hydrogen sulfide as an endogenous relaxation factor in the rat bladder and prostate. Neurourol. Urodyn..

[37] Shibuya N., Koike S., Tanaka M., Ishigami-Yuasa M., Kimura Y., Ogasawara Y., Fukui K., Nagahara N., Kimura H. (2013). A novel pathway for the production of hydrogen sulfide from D-cysteine in mammalian cells. Nat. Commun..

[38] Hildebrandt T.M., Grieshaber M.K. (2008). Three enzymatic activities catalyze the oxidation of sulfide to thiosulfate in mammalian and invertebrate mitochondria. FEBS J..

[39] Libiad M., Yadav P.K., Vitvitsky V., Martinov M., Banerjee R. (2014). Organization of the Human Mitochondrial Hydrogen Sulfide Oxidation Pathway. J. Biol. Chem..

[40] Olson K.R. (2018). H_2_S and polysulfide metabolism: Conventional and unconventional pathways. Biochem. Pharmacol..

[41] Wang Y., Ni X., Chadha R., McCartney C., Lam Y., Brummett B., Ramush G., Xian M. (2022). Methods for Suppressing Hydrogen Sulfide in Biological Systems. Antioxid. Redox Signal..

[42] Pan L., Qin M., Liu X., Zhu Y. (2017). The role of hydrogen sulfide on cardiovascular homeostasis: An overview with update on im-munomodulation. Front. Pharmacol..

[43] Zhang Y., Jing M., Cai C., Zhu S., Zhang C., Wang Q., Zhai Y., Ji X., Wu D. (2023). Role of hydrogen sulphide in physiological and pathological angiogenesis. Cell Prolif..

[44] Módis K., Wolanska K., Vozdek R. (2013). Hydrogen sulfide in cell signaling, signal transduction, cellular bioenergetics and physiology in *C. elegans*. Gen. Physiol. Biophys..

[45] Sunzini F., De Stefano S., Chimenti M.S., Melino S. (2020). Hydrogen Sulfide as Potential Regulatory *Gasotransmitter* in Arthritic Diseases. Int. J. Mol. Sci..

[46] Hughes M.N., Centelles M.N., Moore K.P. (2009). Making and working with hydrogen sulfide: The chemistry and generation of hy-drogen sulfide in vitro and its measurement in vivo: A review. Free Radic. Biol. Med..

[47] Srilatha B., Adaikan P.G., Li L., Moore P.K. (2007). Hydrogen Sulphide: A Novel Endogenous Gasotransmitter Facilitates Erectile Function. J. Sex. Med..

[48] Gai J.-W., Wahafu W., Guo H., Liu M., Wang X.-C., Xiao Y.-X., Zhang L., Xin Z.-C., Jin J. (2013). Further evidence of endogenous hydrogen sulphide as a mediator of relaxation in human and rat bladder. Asian J. Androl..

[49] Guo H., Gai J.-W., Wang Y., Jin H.-F., Du J.-B., Jin J. (2012). Characterization of Hydrogen Sulfide and Its Synthases, Cystathionine β-Synthase and Cystathionine γ-Lyase, in Human Prostatic Tissue and Cells. Urology.

[50] Ghasemi M., Dehpour A.R., Moore K.P., Mani A.R. (2012). Role of endogenous hydrogen sulfide in neurogenic relaxation of rat corpus cavernosum. Biochem. Pharmacol..

[51] Zhang Y., Yang J., Wang T., Wang S.-G., Liu J.-H., Yin C.-P., Ye Z.-Q. (2016). Decreased Endogenous Hydrogen Sulfide Generation in Penile Tissues of Diabetic Rats with Erectile Dysfunction. J. Sex. Med..

[52] Yetik-Anacak G., Dikmen A., Coletta C., Mitidieri E., Dereli M., Donnarumma E., di Villa Bianca R.D.E., Sorrentino R. (2016). Hydrogen sulfide compensates nitric oxide deficiency in murine corpus cavernosum. Pharmacol. Res..

[53] Aydinoglu F., Dalkir F.T., Demirbag H.O., Ogulener N. (2017). The interaction of l-cysteine/H_2_S pathway and muscarinic acetylcholine receptors (mAChRs) in mouse corpus cavernosum. Nitric Oxide.

[54] Zhao K., Li S., Wu L., Lai C., Yang G. (2014). Hydrogen Sulfide Represses Androgen Receptor Transactivation by Targeting at the Second Zinc Finger Module. J. Biol. Chem..

[55] Li G., Xie Z.-Z., Chua J.M., Wong P., Bian J. (2015). Hydrogen sulfide protects testicular germ cells against heat-induced injury. Nitric Oxide.

[56] Gao D.-D., Xu J.-W., Qin W.-B., Peng L., Qiu Z.-E., Wang L.-L., Lan C.-F., Cao X.-N., Xu J.-B., Zhu Y.-X. (2018). Cellular Mechanism Underlying Hydrogen Sulfide Mediated Epithelial K^+^ Secretion in Rat Epididymis. Front. Physiol..

[57] Patel P., Vatish M., Heptinstall J., Wang R., Carson R.J. (2009). The endogenous production of hydrogen sulphide in intrauterine tissues. Reprod. Biol. Endocrinol..

[58] Hydrogen Sulfide Producing Enzymes in Pregnancy and Preeclampsia. https://pubmed.ncbi.nlm.nih.gov/22391326/.

[59] Hu T.-X., Guo X., Wang G., Gao L., He P., Xia Y., Gu H., Ni X. (2017). Mir133b is involved in endogenous hydrogen sulfide suppression of sFlt-1 production in human placenta. Placenta.

[60] Wang K., Ahmad S., Cai M., Rennie J., Fujisawa T., Crispi F., Baily J., Miller M.R., Cudmore M., Hadoke P.W.F. (2013). Ahmed, Dysregulation of hydrogen sulfide producing enzyme cystathionine γ-lyase contributes to maternal hypertension and placental abnormalities in preeclampsia. Circulation.

[61] Cindrova-Davies T., Herrera E.A., Niu Y., Kingdom J., Giussani D.A., Burton G.J. (2013). Reduced Cystathionine γ-Lyase and Increased miR-21 Expression Are Associated with Increased Vascular Resistance in Growth-Restricted Pregnancies: Hydrogen Sulfide as a Placental Vasodilator. Am. J. Pathol..

[62] Sheibani L., Lechuga T.J., Zhang H., Hameed A., Wing D.A., Kumar S., Rosenfeld C.R., Chen D.-B. (2017). Augmented H_2_S production via cystathionine-beta-synthase upregulation plays a role in pregnancy-associated uterine vasodilation. Biol. Reprod..

[63] Lechuga T.J., Qi Q.-R., Magness R.R., Chen D.-B. (2019). Ovine uterine artery hydrogen sulfide biosynthesis in vivo: Effects of ovarian cycle and pregnancy. Biol. Reprod..

[64] Qi Q.-R., Lechuga T.J., Patel B., Nguyen N.A., Yang Y.-H., Li Y., Sarnthiyakul S., Zhang Q.-W., Bai J., Makhoul J. (2020). Enhanced Stromal Cell CBS-H_2_S Production Promotes Estrogen-Stimulated Human Endometrial Angiogenesis. Endocrinology.

[65] Sun Q., Huang J., Yue Y.-J., Xu J.-B., Jiang P., Yang D.-L., Zeng Y., Zhou W.-L. (2016). Hydrogen Sulfide Facilitates Vaginal Lubrication by Activation of Epithelial ATP-Sensitive K+ Channels and Cystic Fibrosis Transmembrane Conductance Regulator. J. Sex. Med..

[66] Dean R.C., Lue T.F. (2005). Physiology of Penile Erection and Pathophysiology of Erectile Dysfunction. Urol. Clin. N. Am..

[67] Jupiter R.C., Yoo D., Pankey E.A., Reddy V.V.G., Edward J.A., Polhemus D.J., Peak T.C., Katakam P., Kadowitz P.J. (2015). Analysis of erectile responses to H_2_S donors in the anesthetized rat. Am. J. Physiol. Circ. Physiol..

[68] Yilmaz E., Kaya-Sezginer E., Yilmaz-Oral D., Cengiz T., Bayatli N., Gur S. (2019). Effects of hydrogen sulphide donor, sodium hy-drosulphide treatment on the erectile dysfunction in l-name-induced hypertensive rats. Andrologia.

[69] Zhong L., Ding W., Zeng Q., He B., Zhang H., Wang L., Fan J., He S., Zhang Y., Wei A. (2020). Sodium tanshinone iia sulfonate attenuates erectile dysfunction in rats with hyperlipidemia. Oxid Med. Cell Longev..

[70] La Fuente J.M., Sevilleja-Ortiz A., García-Rojo E., El Assar M., Fernández A., Pepe-Cardoso A.J., Martínez-Salamanca J.I., Romero-Otero J., Rodríguez-Mañas L., Angulo J. (2020). Erectile dysfunction is associated with defective L-cysteine/hydrogen sulfide pathway in human corpus cavernosum and penile arteries. Eur. J. Pharmacol..

[71] Yetik-Anacak G., Dereli M.V., Sevin G., Ozzayım O., Erac Y., Ahmed A. (2015). Resveratrol Stimulates Hydrogen Sulfide (H_2_S) Formation to Relax Murine Corpus Cavernosum. J. Sex. Med..

[72] Salonia A., Burnett A.L., Graefen M., Hatzimouratidis K., Montorsi F., Mulhall J.P., Stief C. (2012). Prevention and Management of Postprostatectomy Sexual Dysfunctions Part 2: Recovery and Preservation of Erectile Function, Sexual Desire, and Orgasmic Function. Eur. Urol..

[73] Cirino G., Sorrentino R., di Villa Bianca R.D., Popolo A., Palmieri A., Imbimbo C., Fusco F., Longo N., Tajana G., Ignarro L.J. (2003). Involvement of beta 3-adrenergic receptor activation via cyclic gmp- but not no-dependent mechanisms in human corpus cavernosum function. Proc. Natl. Acad. Sci. USA.

[74] Mitidieri E., Tramontano T., Gurgone D., Imbimbo C., Mirone V., Fusco F., Cirino G., di Villa Bianca R.D.E., Sorrentino R. (2017). Β(3) adrenergic receptor activation relaxes human corpus cavernosum and penile artery through a hydrogen sul-fide/cgmp-dependent mechanism. Pharmacol. Res..

[75] Gur S., Peak T., Yafi F.A., Kadowitz P.J., Sikka S.C., Hellstrom W.J.G. (2016). Mirabegron causes relaxation of human and rat corpus cavernosum: Could it be a potential therapy for erectile dysfunction?. BJU Int..

[76] Dayar E., Kara E., Yetik-Anacak G., Hocaoglu N., Bozkurt O., Gidener S., Durmus N. (2018). Do penile haemodynamics change in the presence of hydrogen sulphide (H_2_S) donor in metabolic syndrome-induced erectile dysfunction?. Andrologia.

[77] Qabazard B., Yousif M., Mousa A., Phillips O.A. (2021). GYY4137 attenuates functional impairment of corpus cavernosum and reduces fibrosis in rats with STZ-induced diabetes by inhibiting the TGF-β1/Smad/CTGF pathway. Biomed. Pharmacother..

[78] Yilmaz-Oral D., Kaya-Sezginer E., Oztekin C.V., Bayatli N., Lokman U., Gur S. (2020). Evaluation of combined therapeutic effects of hydrogen sulfide donor sodium hydrogen sulfide and phosphodiesterase type-5 inhibitor tadalafil on erectile dysfunction in a partially bladder outlet obstructed rat model. Neurourol. Urodyn..

[79] Sullivan M.E., Thompson C.S., Dashwood M.R., Khan M.A., Jeremy J.Y., Morgan R.J., Mikhailidis D.P. (1999). Nitric oxide and penile erection: Is erectile dysfunction another manifestation of vascular disease?. Cardiovasc. Res..

[80] Murad F. (2008). Nitric oxide and cyclic guanosine monophosphate signaling in the eye. Can. J. Ophthalmol..

[81] Meng J., Adaikan P.G., Srilatha B. (2013). Hydrogen sulfide promotes nitric oxide production in corpus cavernosum by en-hancing expression of endothelial nitric oxide synthase. Int. J. Impot. Res..

[82] Mostafa T., Rashed L., Nabil N., Abo-Sief A.F., Mohamed M.M., Omar M.S. (2019). Cavernosal hydrogen sulfide levels are associated with nitric oxide and hemeoxygenase levels in diabetic rats. Int. J. Impot. Res..

[83] Olivencia M.A., Esposito E., Brancaleone V., Castaldo S., Cirino G., Pérez-Vizcaino F., Sorrentino R., di Villa Bianca R.D.E., Mitidieri E. (2023). Hydrogen sulfide regulates the redox state of soluble guanylate cyclase in cse(-/-) mice corpus cavernosum microcirculation. Pharmacol. Res..

[84] Elmoneim H.A., Sharabi F., El Din M.M., Louedec L., Norel X., Senbel A. (2017). Potassium channels modulate the action but not the synthesis of hydrogen sulfide in rat corpus cavernosum. Life Sci..

[85] Qabazard B., Yousif M.H.M., Phillips O.A. (2019). Alleviation of impaired reactivity in the corpus cavernosum of STZ-diabetic rats by slow-release H_2_S donor GYY4137. Int. J. Impot. Res..

[86] Aydinoglu F., Belli E.Z.A., Lmaz-Oral D.Y., Ogulener N. (2019). Involvement of rhoa/rho-kinase in l-cysteine/H_2_S pathway-induced inhibition of agonist-mediated corpus cavernosal smooth muscle contraction. Nitric Oxide Biol. Chem..

[87] Fukami K., Sekiguchi F., Yasukawa M., Asano E., Kasamatsu R., Ueda M., Yoshida S., Kawabata A. (2015). Functional upregulation of the H_2_S/Cav3.2 channel pathway accelerates secretory function in neuroendocrine-differentiated human prostate cancer cells. Biochem. Pharmacol..

[88] Zhao Y., Zhang W.-D., Liu X.-Q., Zhang P.-F., Hao Y.-N., Li L., Chen L., Shen W., Tang X.-F., Min L.-J. (2016). Hydrogen Sulfide and/or Ammonia Reduces Spermatozoa Motility through AMPK/AKT Related Pathways. Sci. Rep..

[89] Li S., Ma Y., Ye S., Su Y., Hu D., Xiao F. (2022). Endogenous hydrogen sulfide counteracts polystyrene nanoplastics-induced mito-chondrial apoptosis and excessive autophagy via regulating nrf2 and pgc-1α signaling pathway in mouse spermatocyte-derived gc-2spd(ts) cells. Food Chem. Toxicol. Int. J. Publ. Br. Ind. Biol. Res. Assoc..

[90] Bozkurt M., Degirmentepe R., Polat E., Yildirim F., Sonmez K., Cekmen M., Eraldemir C., Otunctemur A. (2020). Protective effect of hydrogen sulfide on experimental testicular ischemia reperfusion in rats. J. Pediatr. Urol..

[91] Wang J., Shen T., Hong R., Tang S., Zhao X. (2021). H_2_S catalysed by CBS regulates testosterone synthesis through affecting the sulfhydrylation of PDE. J. Cell. Mol. Med..

[92] Wang J., Wang W., Li S., Han Y., Zhang P., Meng G., Xiao Y., Xie L., Wang X., Sha J. (2018). Hydrogen Sulfide As a Potential Target in Preventing Spermatogenic Failure and Testicular Dysfunction. Antioxidants Redox Signal..

[93] Pintus E., Jovičić M., Kadlec M., Ros-Santaella J.L. (2020). Divergent effect of fast- and slow-releasing H_2_S donors on boar spermatozoa under oxidative stress. Sci. Rep..

[94] Xia Y.-Q., Ning J.-Z., Cheng F., Yu W.-M., Rao T., Ruan Y., Yuan R., Du Y. (2019). GYY4137 a H2S donor, attenuates ipsilateral epididymis injury in experimentally varicocele-induced rats via activation of the PI3K/Akt pathway. Iran. J. Basic Med. Sci..

[95] You X.-J., Xu C., Lu J.-Q., Zhu X.-Y., Gao L., Cui X.-R., Li Y., Gu H., Ni X. (2011). Expression of Cystathionine β-synthase and Cystathionine γ-lyase in Human Pregnant Myometrium and Their Roles in the Control of Uterine Contractility. PLoS ONE.

[96] Chen Z., Zhang M., Zhao Y., Xu W., Xiang F., Li X., Zhang T., Wu R., Kang X. (2021). Hydrogen sulfide contributes to uterine quiescence through inhibition of nlrp3 inflammasome activation by suppressing the tlr4/nf-κb signalling pathway. J. Inflamm. Res..

[97] Liu W., Xu C., You X., Olson D.M., Chemtob S., Gao L., Ni X. (2016). Hydrogen Sulfide Delays LPS-Induced Preterm Birth in Mice via Anti-Inflammatory Pathways. PLoS ONE.

[98] Lechuga T.J., Zhang H.-H., Sheibani L., Karim M., Jia J., Magness R.R., Rosenfeld C.R., Chen D.-B. (2015). Estrogen Replacement Therapy in Ovariectomized Nonpregnant Ewes Stimulates Uterine Artery Hydrogen Sulfide Biosynthesis by Selectively Up-Regulating Cystathionine β-Synthase Expression. Endocrinology.

[99] Lechuga T.J., Bilg A.K., Patel B.A., Nguyen N.A., Qi Q., Chen D. (2019). Estradiol-17β stimulates H_2_S biosynthesis by er-dependent cbs and cse transcription in uterine artery smooth muscle cells in vitro. J. Cell Physiol..

[100] Lei S., Cao Y., Sun J., Li M., Zhao D. (2018). H_2_S promotes proliferation of endometrial stromal cells via activating the nf-κb pathway in endometriosis. Am. J. Transl. Res..

[101] Guzmán M.A., Navarro M.A., Carnicer R., Sarría A.J., Acín S., Arnal C., Muniesa P., Surra J.C., Arbonés-Mainar J.M., Maeda N. (2006). Cystathionine beta-synthase is essential for female reproductive function. Hum. Mol. Genet..

[102] Sarno L., Raso G.M., Bianca R.D.d.V., Mitidieri E., Maruotti G., Esposito G., Meli R., Sorrentino R., Martinelli P. (2012). OS064. Contribute of the L-cysteine/H_2_S pathway in placenta homeostasisin hypertensive disorders. Pregnancy Hypertens..

[103] Hu T., Wang G., Zhu Z., Huang Y., Gu H., Ni X. (2015). Increased ADAM10 expression in preeclamptic placentas is associated with decreased expression of hydrogen sulfide production enzymes. Placenta.

[104] Du J., Wang P., Gou Q., Jin S., Xue H., Li D., Tian D., Sun J., Zhang X., Teng X. (2022). Hydrogen sulfide ameliorated preeclampsia via suppression of toll-like receptor 4-activated inflammation in the rostral ventrolateral medulla of rats. Biomed. Pharmacother..

[105] Hu T.-X., Wang G., Guo X.-J., Sun Q.-Q., He P., Gu H., Huang Y., Gao L., Ni X. (2016). MiR 20a,-20b and -200c are involved in hydrogen sulfide stimulation of VEGF production in human placental trophoblasts. Placenta.

[106] Chen D.-B., Feng L., Hodges J.K., Lechuga T.J., Zhang H. (2017). Human trophoblast-derived hydrogen sulfide stimulates placental artery endothelial cell angiogenesis. Biol. Reprod..

[107] Xu J.-W., Gao D.-D., Peng L., Qiu Z.-E., Ke L.-J., Zhu Y.-X., Zhang Y.-L., Zhou W.-L. (2019). The gasotransmitter hydrogen sulfide inhibits transepithelial anion secretion of pregnant mouse endometrial epithelium. Nitric Oxide.

[108] Lu L., Kingdom J., Burton G.J., Cindrova-Davies T. (2017). Placental stem villus arterial remodeling associated with reduced hydrogen sulfide synthesis contributes to human fetal growth restriction. Am. J. Pathol..

[109] Wang B., Xu T., Li Y., Wang W., Lyu C., Luo D., Yang Q., Ning N., Chen Z.-J., Yan J. (2020). Trophoblast H_2_S Maintains Early Pregnancy via Regulating Maternal-Fetal Interface Immune Hemostasis. J. Clin. Endocrinol. Metab..

[110] Zhao F., Lei F., Yan X., Zhang S., Wang W., Zheng Y. (2018). Protective effects of hydrogen sulfide against cigarette smoke expo-sure-induced placental oxidative damage by alleviating redox imbalance via nrf2 pathway in rats. Cell. Physiol. Biochem. Int. J. Exp. Cell. Physiol. Biochem. Pharmacol..

[111] Wu W., Tan Q.-Y., Xi F.-F., Ruan Y., Wang J., Luo Q., Dou X.-B., Hu T.-X. (2022). NLRP3 inflammasome activation in gestational diabetes mellitus placentas is associated with hydrogen sulfide synthetase deficiency. Exp. Ther. Med..

[112] Liang R., Yu W., Du J., Yang L., Yang J., Xu J., Shang M., Guo J. (2007). Cystathionine beta synthase participates in murine oocyte maturation mediated by homocysteine. Reprod. Toxicol..

[113] Bucci M., Papapetropoulos A., Vellecco V., Zhou Z., Pyriochou A., Roussos C., Roviezzo F., Brancaleone V., Cirino G. (2010). Hydrogen Sulfide Is an Endogenous Inhibitor of Phosphodiesterase Activity. Arterioscler. Thromb. Vasc. Biol..

[114] La Fuente J.M., Fernández A., Pepe-Cardoso A.J., Martínez-Salamanca J.I., Louro N., Angulo J. (2019). L-cysteine/hydrogen sulfide pathway induces cgmp-dependent relaxation of corpus cavernosum and penile arteries from patients with erectile dysfunction and improves arterial vasodilation induced by pde5 inhibition. Eur. J. Pharmacol..

[115] Nelson M.T., Quayle J.M. (1995). Physiological roles and properties of potassium channels in arterial smooth muscle. Am. J. Physiol..

[116] Christ G.J., Spray D.C., Brink P.R. (1993). Characterization of K Currents in Cultured Human Corporal Smooth Muscle Cells. J. Androl..

[117] Lee S., Lee C.O. (2005). Inhibition of Na^+^-K^+^ pump and l-type Ca^2+^ channel by glibenclamide in guinea pig ventricular myocytes. J. Pharmacol. Exp. Ther..

[118] Chitaley K., Wingard C.J., Webb R.C., Branam H., Stopper V.S., Lewis R.W., Mills T.M. (2001). Antagonism of rho-kinase stim-ulates rat penile erection via a nitric oxide-independent pathway. Nat. Med..

[119] Aydinoglu F., Ogulener N. (2017). The role of arachidonic acid/cyclooxygenase cascade, phosphodiesterase iv and rho-kinase in H_2_S-induced relaxation in the mouse corpus cavernosum. Pharmacol. Rep. PR.

[120] Ngowi E.E., Afzal A., Sarfraz M., Khattak S., Zaman S.U., Khan N.H., Li T., Jiang Q.-Y., Zhang X., Duan S.-F. (2021). Role of hydrogen sulfide donors in cancer development and progression. Int. J. Biol. Sci..

[121] Chattopadhyay M., Kodela R., Nath N., Dastagirzada Y.M., Velázquez-Martínez C.A., Boring D., Kashfi K. (2012). Hydrogen sul-fide-releasing nsaids inhibit the growth of human cancer cells: A general property and evidence of a tissue type-independent effect. Biochem. Pharmacol..

[122] Bigagli E., Luceri C., De Angioletti M., Chegaev K., D’ambrosio M., Riganti C., Gazzano E., Saponara S., Longini M., Luceri F. (2018). New NO- and H2S-releasing doxorubicins as targeted therapy against chemoresistance in castration-resistant prostate cancer: In vitro and in vivo evaluations. Investig. New Drugs.

[123] Zhou B., Jia B.-X., Zhang M.-J., Tan Y.-J., Liang W.-Y., Gan X., Li H.-T., Yang X., Shen X.-C. (2023). Zn2+-interference and H_2_S-mediated gas therapy based on ZnS-tannic acid nanoparticles synergistic enhancement of cell apoptosis for specific treatment of prostate cancer. Colloids Surf. B Biointerfaces.

[124] Zhang W., Zhao Y., Zhang P., Hao Y., Yu S., Min L., Li L., Ma D., Chen L., Yi B. (2018). Decrease in male mouse fertility by hydrogen sulfide and/or ammonia can Be inheritable. Chemosphere.

[125] Moustafa A. (2021). Changes in nitric oxide, carbon monoxide, hydrogen sulfide and male reproductive hormones in response to chronic restraint stress in rats. Free Radic. Biol. Med..

[126] Azarbarz N., Seifabadi Z.S., Moaiedi M.Z., Mansouri E. (2020). Assessment of the effect of sodium hydrogen sulfide (hydrogen sulfide donor) on cisplatin-induced testicular toxicity in rats. Environ. Sci. Pollut. Res..

[127] Ohlander S.J., Lindgren M.C., Lipshultz L.I. (2016). Testosterone and male infertility. Urol. Clin. N. Am..

[128] Shi H., Li Q.-Y., Li H., Wang H.-Y., Fan C.-X., Dong Q.-Y., Pan B.-C., Ji Z.-L., Li J.-Y. (2024). ROS-induced oxidative stress is a major contributor to sperm cryoinjury. Hum. Reprod..

[129] Wiliński B., Wiliński J., Gajda M., Jasek E., Somogyi E., Owacki M.A.G., Liwa L. (2015). Sodium hydrosulfide exerts a transitional at-tenuating effect on spermatozoa migration in vitro. Folia. Biol..

[130] Kadlec M., Pintus E., Ros-Santaella J.L. (2022). The Interaction of NO and H_2_S in Boar Spermatozoa under Oxidative Stress. Animals.

[131] Pintus E., Chinn A.F., Kadlec M., García-Vázquez F.A., Novy P., Matson J.B., Ros-Santaella J.L. (2023). N-thiocarboxyanhydrides, amino acid-derived enzyme-activated H_2_S donors, enhance sperm mitochondrial activity in presence and absence of oxidative stress. BMC Vet. Res..

[132] Jones R. (1996). Murdoch Regulation of the motility and metabolism of spermatozoa for storage in the epididymis of eutherian and marsupial mammals. Reprod. Fertil. Dev..

[133] Lorian K., Kadkhodaee M., Kianian F., Abdi A., Ranjbaran M., Ashabi G., Seifi B. (2020). Long-term nahs administration reduces ox-idative stress and apoptosis in a rat model of left-side varicocele. Andrologia.

[134] Shafie A., Kianian F., Ashabi G., Kadkhodaee M., Ranjbaran M., Hajiaqaei M., Lorian K., Abdi A., Seifi B. (2022). Beneficial effects of combination therapy with testosterone and hydrogen sulfide by reducing oxidative stress and apoptosis: Rat experimental varicocele model. Int. J. Reprod. Biomed. (IJRM).

[135] Hayden L.J., Goeden H., Roth S.H. (1990). Growth and Development in the Rat during Sub-Chronic Exposure to Low Levels of Hydrogen Sulfide. Toxicol. Ind. Health.

[136] Robinson H., Wray S. (2012). A New Slow Releasing, H_2_S Generating Compound, GYY4137 Relaxes Spontaneous and Oxytocin-Stimulated Contractions of Human and Rat Pregnant Myometrium. PLoS ONE.

[137] Mitidieri E., Tramontano T., Donnarumma E., Brancaleone V., Cirino G., Bianca R.D.d.V., Sorrentino R. (2016). l -Cys/CSE/H 2 S pathway modulates mouse uterus motility and sildenafil effect. Pharmacol. Res..

[138] Hu R., Lu J., You X., Zhu X., Hui N., Ni X. (2011). Hydrogen sulfide inhibits the spontaneous and oxytocin-induced contractility of human pregnant myometrium. Gynecol. Endocrinol. Off. J. Int. Soc. Gynecol. Endocrinol..

[139] Challis J.R., Lockwood C.J., Myatt L., Norman J.E., Strauss J.F.R., Petraglia F. (2009). Inflammation and pregnancy. Reprod. Sci..

[140] Osman I., Young A., Ledingham M.A., Thomson A.J., Jordan F., Greer I.A., Norman J.E. (2003). Leukocyte density and pro-inflammatory cytokine expression in human fetal membranes, decidua, cervix and myometrium before and during labour at term. Mol. Hum. Reprod..

[141] Ray A., Chakraborty K., Ray P. (2013). Immunosuppressive MDSCs induced by TLR signaling during infection and role in resolution of inflammation. Front. Cell. Infect. Microbiol..

[142] Chen D., Zheng J. (2014). Regulation of Placental Angiogenesis. Microcirculation.

[143] Das A., Mantena S.R., Kannan A., Evans D.B., Bagchi M.K., Bagchi I.C. (2009). De novo synthesis of estrogen in pregnant uterus is critical for stromal decidualization and angiogenesis. Proc. Natl. Acad. Sci. USA.

[144] Loriaux D.L., Ruder H.J., Knab D.R., Lipsett M.B. (1972). Estrone sulfate, estrone, estradiol and estriol plasma levels in human preg-nancy. J. Clin. Endocrinol. Metab..

[145] Bai J., Lechuga T.J., Makhoul J., Yan H., Major C., Hameed A., Chen D. (2023). Erα/erβ-directed cbs transcription mediates e2β-stimulated huaec H_2_S production. J. Mol. Endocrinol..

[146] Maybin J.A., Critchley H.O. (2015). Menstrual physiology: Implications for endometrial pathology and beyond. Hum. Reprod. Updat..

[147] Ruan Y.C., Guo J.H., Liu X., Zhang R., Tsang L.L., Da Dong J., Chen H., Yu M.K., Jiang X., Zhang X.H. (2012). Activation of the epithelial Na^+^ channel triggers prostaglandin E2 release and production required for embryo implantation. Nat. Med..

[148] Ajonuma L.C., Ng E.H.Y., Chan H.C. (2002). New insights into the mechanisms underlying hydrosalpinx fluid formation and its ad-verse effect on ivf outcome. Hum. Reprod. Update.

[149] Ahmad S., Ahmed A. (2004). Elevated placental soluble vascular endothelial growth factor receptor-1 inhibits angiogenesis in preeclampsia. Circ. Res..

[150] Ahmed A. (2014). Molecular mechanisms and therapeutic implications of the carbon monoxide/hmox1 and the hydrogen sulfide/CSE pathways in the prevention of pre-eclampsia and fetal growth restriction. Pregnancy Hypertens..

[151] Arbeille P. (1997). Fetal arterial doppler-iugr and hypoxia. Eur. J. Obstet. Gynecol. Reprod. Biol..

[152] Raghupathy R. (1997). Th 1-type immunity is incompatible with successful pregnancy. Immunol. Today.

[153] La Rocca C., Carbone F., Longobardi S., Matarese G. (2014). The immunology of pregnancy: Regulatory T cells control maternal immune tolerance toward the fetus. Immunol. Lett..

[154] Yang R., Qu C., Zhou Y., Konkel J.E., Shi S., Liu Y., Chen C., Liu S., Liu D., Chen Y. (2015). Hydrogen Sulfide Promotes Tet1- and Tet2-Mediated Foxp3 Demethylation to Drive Regulatory T Cell Differentiation and Maintain Immune Homeostasis. Immunity.

[155] Chen W., Jin W., Hardegen N., Lei K., Li L., Marinos N., Mcgrady G., Wahl S.M. (2003). Conversion of peripheral cd4+cd25- naive t cells to cd4+cd25+ regulatory t cells by tgf-beta induction of transcription factor foxp3. J. Exp. Med..

[156] Chavan A.R., Griffith O.W., Wagner G.P. (2017). The inflammation paradox in the evolution of mammalian pregnancy: Turning a foe into a friend. Curr. Opin. Genet. Dev..

[157] Wegmann T.G., Lin H., Guilbert L., Mosmann T.R. (1993). Bidirectional cytokine interactions in the maternal-fetal relationship: Is successful pregnancy a th2 phenomenon?. Immunol. Today.

[158] Singh N., Prasad P., Kumar P., Singh L.C., Das B., Rastogi S. (2015). Does aberrant expression of cyclooxygenase-2 and prostaglandin-E2 receptor genes lead to abortion in *Chlamydia trachomatis*-infected women. J. Matern. Neonatal Med..

[159] Wang Y., Zhao A.-M., Lin Q.-D. (2010). Role of cyclooxygenase-2 signaling pathway dysfunction in unexplained recurrent spontaneous abortion. Chin. Med. J..

[160] Schumacher A. (2017). Human chorionic gonadotropin as a pivotal endocrine immune regulator initiating and preserving fetal tol-erance. Int. J. Mol. Sci..

[161] Schumacher A., Heinze K., Witte J., Poloski E., Linzke N., Woidacki K., Zenclussen A.C. (2013). Human Chorionic Gonadotropin as a Central Regulator of Pregnancy Immune Tolerance. J. Immunol..

[162] Ban Y., Chang Y., Dong B., Kong B., Qu X. (2013). Indoleamine 2,3-dioxygenase levels at the normal and recurrent spontaneous abortion fetal-maternal interface. J. Int. Med. Res..

[163] Munn D.H., Zhou M., Attwood J.T., Bondarev I., Conway S.J., Marshall B., Brown C., Mellor A.L. (1998). Prevention of Allogeneic Fetal Rejection by Tryptophan Catabolism. Science.

[164] Frumento G., Rotondo R., Tonetti M., Damonte G., Benatti U., Ferrara G.B. (2002). Tryptophan-derived Catabolites Are Responsible for Inhibition of T and Natural Killer Cell Proliferation Induced by Indoleamine 2,3-Dioxygenase. J. Exp. Med..

[165] Guo P.-F., Du M.-R., Wu H.-X., Lin Y., Jin L.-P., Li D.-J. (2010). Thymic stromal lymphopoietin from trophoblasts induces dendritic cell–mediated regulatory TH2 bias in the decidua during early gestation in humans. Blood.

[166] Velicky P., Knöfler M., Pollheimer J. (2016). Function and control of human invasive trophoblast subtypes: Intrinsic vs. maternal control. Cell Adhes. Migr..

[167] Einarson A., Riordan S. (2009). Smoking in pregnancy and lactation: A review of risks and cessation strategies. Eur. J. Clin. Pharmacol..

[168] Jauniaux E., Burton G.J. (2007). Morphological and biological effects of maternal exposure to tobacco smoke on the feto-placental unit. Early Hum. Dev..

[169] Olmos-Ortiz A., Flores-Espinosa P., Díaz L., Velázquez P., Ramírez-Isarraraz C., Zaga-Clavellina V. (2021). Immunoendocrine Dysregulation during Gestational Diabetes Mellitus: The Central Role of the Placenta. Int. J. Mol. Sci..

[170] Estienne A., Portela V.M., Choi Y., Zamberlam G., Boerboom D., Roussel V., Meinsohn M.-C., Brännström M., Curry T.E., Jo M. (2019). The endogenous hydrogen sulfide generating system regulates ovulation. Free. Radic. Biol. Med..

[171] Fontes P.K., dos Santos E.C., da Rocha H.C., de Lima C.B., Milazzotto M.P. (2024). Metabolic stressful environment drives epigenetic modifications in oviduct epithelial cells. Theriogenology.

[172] Jeremy J.Y., Jones R.A., Koupparis A.J., Hotston M., Persad R., Angelini G.D., Shukla N. (2007). Reactive oxygen species and erectile dysfunction: Possible role of NADPH oxidase. Int. J. Impot. Res..

[173] Jeremy J.Y., Rowe D., Emsley A.M., Newby A.C. (1999). Nitric oxide and the proliferation of vascular smooth muscle cells. Cardiovasc. Res..

[174] Koupparis A.J., Jeremy J.Y., Muzaffar S., Persad R., Shukla N. (2005). Sildenafil inhibits the formation of superoxide and the expression of gp47 nad[p]h oxidase induced by the thromboxane a2 mimetic, u46619, in corpus cavernosal smooth muscle cells. BJU Int..

[175] Shukla N., Jones R., Persad R., Angelini G.D., Jeremy J.Y. (2005). Effect of sildenafil citrate and a nitric oxide donating sildenafil derivative, NCX 911, on cavernosal relaxation and superoxide formation in hypercholesterolaemic rabbits. Eur. J. Pharmacol..

[176] Muzaffar S., Shukla N., Bond M., Sala-Newby G., Angelini G.D., Newby A.C., Jeremy J.Y. (2008). Acute inhibition of superoxide for-mation and rac1 activation by nitric oxide and iloprost in human vascular smooth muscle cells in response to the thromboxane a2 analogue, u46619. Prostaglandins Leukot. Essent. Fat. Acids.

[177] Wan S., Shukla N., Angelini G.D., Yim A.P.C., Johnson J.L., Jeremy J.Y. (2007). Nitric oxide-donating aspirin (ncx 4016) inhibits neoin-timal thickening in a pig model of saphenous vein-carotid artery interposition grafting: A comparison with aspirin and mor-pholinosydnonimine (sin-1). J. Thorac. Cardiovasc. Surg..

[178] Hotston M., Jeremy J.Y., Bloor J., Greaves N.S., Persad R., Angelini G., Shukla N. (2008). Homocysteine and copper interact to promote type 5 phosphodiesterase expression in rabbit cavernosal smooth muscle cells. Asian J. Androl..

[179] Muzaffar S., Jeremy J.Y., Angelini G.D., Stuart-Smith K., Shukla N. (2003). Role of the endothelium and nitric oxide synthases in modulating superoxide formation induced by endotoxin and cytokines in porcine pulmonary arteries. Thorax.

[180] Muzaffar S., Shukla N., Bond M., Newby A.C., Angelini G.D., Sparatore A., Del Soldato P., Jeremy J.Y. (2008). Exogenous Hydrogen Sulfide Inhibits Superoxide Formation, NOX-1 Expression and Rac1 Activity in Human Vascular Smooth Muscle Cells. J. Vasc. Res..

[181] Bibli S., Yang G., Zhou Z., Wang R., Topouzis S., Papapetropoulos A. (2015). Role of cgmp in hydrogen sulfide signaling. Nitric Oxide Biol. Chem..

[182] Song Y., Qu Y., Mao C., Zhang R., Jiang D., Sun X. (2023). Post-translational modifications of Keap1: The state of the art. Front. Cell Dev. Biol..

[183] Mustafa A.K., Sikka G., Gazi S.K., Steppan J., Jung S.M., Bhunia A.K., Barodka V.M., Gazi F.K., Barrow R.K., Wang R. (2011). Hydrogen Sulfide as Endothelium-Derived Hyperpolarizing Factor Sulfhydrates Potassium Channels. Circ. Res..

[184] Sun Y., Huang Y., Yu W., Chen S., Yao Q., Zhang C., Bu D., Tang C., Du J., Jin H. (2017). Sulfhydration-associated phosphodiesterase 5A dimerization mediates vasorelaxant effect of hydrogen sulfide. Oncotarget.

